# HIV protein Nef expression in human microglia drives the release of distinct Nef-containing extracellular vesicles

**DOI:** 10.20517/evcna.2025.106

**Published:** 2025-12-02

**Authors:** Teja Lavrin, Jure Loboda, Jana Ferdin, Valentina Levak, Simona Sitar, Marija Holcar, Nataša Resnik, Matjaž Stenovec, Alenka Trampuš Bakija, Peter Veranič, Ema Žagar, Magda Tušek Žnidarič, Pia Pužar Dominkuš, Metka Lenassi

**Affiliations:** ^1^Institute of Biochemistry and Molecular Genetics, Faculty of Medicine, University of Ljubljana, Ljubljana 1000, Slovenia.; ^2^Department of Biotechnology and Systems Biology, National Institute of Biology, Ljubljana 1000, Slovenia.; ^3^Jozef Stefan International Postgraduate School, Ljubljana 1000, Slovenia.; ^4^Department of Polymer Chemistry and Technology, National Institute of Chemistry, Ljubljana 1000, Slovenia.; ^5^Institute of Cell Biology, Faculty of Medicine, University of Ljubljana, Ljubljana 1000, Slovenia.; ^6^Celica Biomedical, Ljubljana 1000, Slovenia.; ^7^Laboratory of Neuroendocrinology-Molecular Cell Physiology, Institute of Pathophysiology, Faculty of Medicine, University of Ljubljana, Ljubljana 1000, Slovenia.; ^8^Clinical Institute for Special Laboratory Diagnostics, University Children’s Hospital, University Medical Centre Ljubljana, Ljubljana 1000, Slovenia.

**Keywords:** Human immunodeficiency virus (HIV), microglia, protein Nef, extracellular vesicles (EVs), HIV-associated neurocognitive disorder (HAND)

## Abstract

**Aim:** Human immunodeficiency virus (HIV)-associated neurocognitive disorders (HAND) persist in effectively treated HIV-infected individuals, in part due to HIV reservoirs in brain microglia, which express low levels of viral proteins such as Nef. This study aimed to elucidate how microglia release Nef into the extracellular space, where it exerts its biological functions.

**Methods:** Here, we systematically characterized extracellular particles released from immortalized human microglia (h-microglia) expressing Nef alone or after HIV infection. Importantly, we established a novel h-microglia model harboring a stably integrated Nef tagged with green fluorescent protein (Nef.GFP) transgene under an inducible promoter. Extracellular vesicles (EVs) were enriched from culture media and analyzed for morphology, size, concentration and molecular composition, including Nef content, by (super-resolution) fluorescence microscopy, (immunogold) transmission electron microscopy, asymmetric flow field-flow fractionation coupled to a multi-angle light-scattering detector, nanoparticle tracking analysis, and nano-flow cytometry and immunoblotting.

**Results:** Nef.GFP expression increased particle release up to 11.7-fold compared with controls or known stimulants adenosine triphosphate (ATP) and ionomycin. Compared to the latter, the particles were also significantly smaller (root mean square radius, Rrms = 172 nm) and displayed unique protein and density profiles. All data support the EV nature of the released particles. Approximately half of the Nef.GFP-induced EVs contained Nef (45.5% ± 15.8%), with immunogold labeling confirming its intraluminal localization. Notably, infection with HIV isolates NL4-3 and YU-2 likewise produced Nef-positive EVs distinct from virions.

**Conclusion:** Our findings importantly contribute to understanding the source and characteristics of extracellular Nef in the central nervous system of HIV infected individuals and offer new tools to study HIV Nef biology. Nef-laden EVs should be further investigated as potential therapeutic targets in HAND.

## INTRODUCTION

Human immunodeficiency virus 1 (HIV) infection has transformed over the years from a terminal to a chronic disease, with more than 70% of infected individuals globally maintaining viral suppression with antiretroviral therapy (ART)^[1]^. Still, although people with HIV are living longer, they are disproportionately burdened with aging-related diseases compared to the healthy population, including a 75% increase in the risk of neurocognitive impairment, also known as HIV-associated neurocognitive disorder (HAND)^[2,3]^. Most cases are classified as asymptomatic neurocognitive impairment or mild neurocognitive disorder but can also progress to HIV-associated dementia^[4]^. Central to neuroinflammation and development of HAND are microglia, the resident immune cells of the central nervous system (CNS). Although the exact mechanisms remain unclear, recent data support a direct contribution of latent HIV reservoirs in microglia, which persist even in individuals on effective ART^[5,6]^.

Several recent studies support sustained low-level expression of HIV transcripts and viral proteins even in defective microglia HIV reservoirs, producing replication-incompetent viruses. Single-genome sequencing performed on distinct autopsied tissues of three HIV-infected individuals on ART found that only 3.2% of all HIV proviruses in CNS tissues are intact, which is low compared to other established tissue reservoirs such as lymphoid and gastrointestinal tissues^[7]^. The study also showed that defective HIV proviral sequences are more broadly distributed across the CNS regions compared to intact proviruses^[7]^. Single-cell genome and transcriptome sequencing of microglia isolated from autopsied tissue of HIV-infected individuals on ART further showed that up to 0.5% contain detectable HIV RNA and/or HIV DNA integrated into open chromatin. Microglia with detectable HIV RNA adopt an inflammatory phenotype^[8]^. Interestingly, studies on CD4+ HIV reservoirs show that the timing of ART initiation does not affect the proviral landscape, e.g., the ratio between intact and defective proviruses^[9]^. A separate study demonstrated that defective HIV proviruses generate new protein-coding RNA transcripts in peripheral blood CD4+ T cells of patients at all stages of infection^[10]^. Furthermore, these defective proviruses can be translationally competent and can produce HIV proteins^[11]^, with the presence of the latter correlating with persistent immune activation observed in ART-suppressed individuals^[11,12]^. Importantly, Nef expression has been observed in a CD4+ T cell clone harboring defective provirus^[11]^ and has also been detected in the plasma of approximately half of virally suppressed HIV-infected individuals^[13]^. However, studies on microglial HIV reservoirs and Nef are still missing.

Nef is a key HIV pathogenic factor implicated in neural dysfunction by promoting neuroinflammation through oxygen species production^[14]^, disruption of lipid metabolism^[15,16]^, modulation of autophagy^[17]^ and apoptosis^[18]^, and impairment of myelin integrity^[19]^. Notably, Nef was detected postmortem in the brains of HAND patients^[20]^, as well as in Iba-1-positive microglia and macrophages in the brains of Simian immunodeficiency virus (SIV)-infected macaques on ART^[21]^. *In vitro* studies on microglial cultures showed that Nef modulates activation state, viability and metabolism by increasing proinflammatory chemokines, promoting inflammasome activation, and inducing cellular senescence^[22-24]^. Additionally, our intracellular trafficking study of Nef tagged with green fluorescent protein (Nef.GFP)-expressing human microglia (h-microglia) indicated extracellular release of vesicle-like structures carrying Nef^[25]^. Extracellular secretion of Nef in EVs (Nef EVs) appears to be conserved across different organisms^[26]^, cell types^[27-31]^ and HIV strains^[19,29]^, but has not yet been described in h-microglia. Importantly, data from cellular models^[15,16,26,31]^ and animal models^[19,32,33]^ support a role for Nef EVs in the recognized functions of Nef in the CNS. Still, the basic biology of Nef-containing extracellular vesicles (EVs) released from h-microglia, a major CNS source of HIV transcripts and proteins in ART-treated individuals, remains largely unknown. To bridge this gap in knowledge, we aimed to comprehensively characterize the extracellular release of Nef from h-microglia.

To this end, we systematically characterized extracellular particles released from immortalized microglia isolated from healthy human cerebral cortex (h-microglia^[34]^), expressing Nef alone or during HIV infection. Specifically, h-microglia were transfected with a Nef.GFP plasmid, transduced with a lentiviral vector for stable integration of the Nef.GFP gene under an inducible promoter, or infected with pseudotyped HIV isolates NL4-3 and YU-2. Extracellular particles (including EVs) were enriched from culture media and analyzed for morphology, size, concentration, and molecular composition, including Nef content, by (super-resolution) fluorescence microscopy, (immunogold) transmission electron microscopy (TEM), asymmetric flow field-flow fractionation coupled to multiple detectors, nanoparticle tracking analysis (NTA), nano-flow cytometry, and immunoblotting. Across diverse h‑microglia HIV reservoir models, microglial Nef expression selectively induced the release of EVs with distinct biophysical and molecular signatures that encapsulate Nef.

## METHODS

### Cell lines

Immortalized [simian virus 40 large T antigen (SV40)/human telomerase reverse transcriptase (hTERT)] h-microglia, isolated from fresh CNS cortical tissue, were kindly provided by the Jonathan Karn laboratory (Western Reserve University, Cleveland, USA)^[34]^. Cells were cultivated at 37 °C and 5% CO_2_/95% air in Dulbecco’s Modified Eagle Medium (DMEM, Sigma Aldrich, USA), supplemented with 1 × GlutaMAX^TM^ (Thermo Fisher Scientific, USA), 10% (v/v) heat-inactivated and sterile-filtered Fetal Bovine Serum (FBS, Sigma Aldrich, USA), 10,000 U/mL penicillin and 10 mg/mL streptomycin (Sigma Aldrich, USA). In the case of EV or virion enrichment from culture media, we used EV-depleted FBS, which was prepared by overnight (~20 h) ultracentrifugation at 100,000 × *g* (4 °C) and filtered through a 0.22 µm filter (Merck Millipore, USA). For assays, we used h-microglia between passages 4 and 12 that tested negative for mycoplasma.

Cell viability of h-microglia cultures was assessed by counting the dead cells with TC20 Automated Cell Counter (Bio-Rad, USA), after staining with 0.4% Trypan Blue Solution (ratio 1:1; Gibco, Thermo Fisher Scientific, USA).

The percentage of green fluorescent protein (GFP) or Nef.GFP-expressing cells was assessed by counting green-fluorescent cells with FACSCanto II Flow Cytometer equipped with a blue and red laser, running FACSDiva software (both BD Biosciences). Digital data were analyzed using FlowJo software (Tree Star Inc.). The proportion of dead cells in each experiment never exceeded 5%.

### Plasmids

For transient expression of Nef.GFP, h-microglia were transfected with a plasmid encoding GFP (pEGFP-N1; designated as pGFP; Takara Bio, USA) or Nef protein of the HIV-1 SF2 isolate tagged with GFP (pEGFP-N1-NefSF2; designated as pNef.GFP; BEI Resources Repository, formerly NIH HIV Reagent Program, USA).

For stable expression of Nef.GFP, the Nef.GFP gene from HIV-1 SF2, placed under an inducible TRE3G promoter, was integrated into the genome of h-microglia using lentiviral vectors eLV.EF1.Tet3G-9 (expressing the Tet-On 3G transactivator under the EF1α promoter) and rLV.TRE3G.NefSF2-EGFP.hPGK.Puro (carrying the Nef.GFP gene under the TRE3G promoter; designated LV-Nef.GFP) (Flash Therapeutics, France).

For controls, pGFP was used in transient expression experiments, lentiviral vector rLV.TRE3G.EGFP.hPGK.Puro (expressiong GFP under TRE3G promoter, designated as LV-GFP) in combination with eLV.EF1.Tet3G-9 was used for stable expression.

For production of pseudotyped HIV-1 viral stocks, HEK293T cells were transfected with plasmids encoding the HIV-1 isolate YU-2 (ARP-1350), or molecular clones NL4-3 (ARP-114) and Nef-deleted NL4-3 (NL4-3 Δnef, ARP-12755) (all obtained from the BEI Resources Repository, USA), together with a plasmid encoding vesicular stomatitis virus envelope protein (VSV-G; pCMV-VSV-G; Addgene, USA).

### Approaches towards expressing Nef in h-microglia

#### Transient expression of Nef.GFP

h-microglia cells were grown overnight to reach 80% confluence, after which they were washed with Dulbecco’s phosphate buffered saline (DPBS, Sigma Aldrich, USA), trypsinized (Sigma Aldrich, USA), and resuspended in non-supplemented DMEM. For each reaction, 2.5 × 10^6^ cells were plated in a 10 cm Petri dish. Without changing the medium, a transfection mixture containing 8 µg of plasmid DNA and 25 µL of XtremeGene transfection reagent (Roche, Switzerland), mixed in 575 µL of non-supplemented DMEM, was added dropwise to the cells following the manufacturer’s instructions. After 4 h at 37 °C to allow h-microglia attachment, cells were washed with DPBS and later grown in EV-depleted complete DMEM for 48 h.

#### Stable expression of Nef.GFP

To generate h-microglial cells with an integrated Nef.GFP (or GFP) gene under an inducible promoter, we used a third-generation lentiviral vector combined with the TET-ON inducible gene expression system, which integrates the gene of interest under a doxycycline (DOX)-inducible promoter into the genome of h-microglial cells (Flash Therapeutics, France), following the manufacturer’s instructions for the transduction of adherent cells.

Briefly, h-microglia cells were seeded at a density of 5 × 10^4^ cells per well in a 24-well plate and incubated overnight to reach 80%-90% confluency. To identify the optimal range of efficient transduction with minimal cytotoxicity, we transduced h-microglia with lentiviral vector ILV-EF1α-TurboGFP (expressing TurboGFP under the EF1α promoter) and determined the multiplicity of infection (MOI). The transduction mix was prepared from viral vector (calculated for MOI 20), the infection enhancer polybrene (4 µg/mL) and complete culture media. Sequential double transduction was then performed. First, cells were transduced with the lentiviral vector eLV.EF1.Tet3G-9, enabling constitutive expression of TET-ON 3G transactivator protein (designated as LV-control), and transduced cells were selected with geneticin. Next, cells were further transduced with either LV-Nef.GFP or LV-GFP, both carrying genes under the control of an inducible TRE3G promoter. Four hours after each transduction, cells were washed with DPBS and fresh complete medium was added. Stable h-microglia cells with integrated Nef.GFP or GFP gene were selected using puromycin.

To assess critical concentration of DOX for h-microglia high viability, combined with efficient induction of gene expression, cells were seeded in 24-well plates overnight to reach 60%-70% confluence. After washing with DPBS, EV-depleted complete DMEM supplemented with 0-500 ng/mL DOX was added, and cells were cultured for 48 h. Cell viability and Nef.GFP expression were analyzed as described above (see Section Cell lines). We determined that a DOX concentration of 50 ng/mL effectively induces gene expression with low toxicity for h-microglia.

#### Nef expression in the context of HIV-1 infection

Pseudotyped HIV-1 viral stocks were prepared from 72 h old cultures of HEK293T, co-transfected with HIV-1 (isolate YU-2 or clones NL4-3 and NL4-3 Δnef) and pCMV-VSV-G coding plasmids. Transfections were performed using XtremeGENE HP DNA Transfection Reagent (Roche, Switzerland), according to the manufacturer’s instructions. Precleared culture media of transfected cells were filtered through a 0.22 μm filter and viral particles were concentrated using Amicon Ultra-15 filters (Merck Millipore, USA), aliquoted, and stored at -80 °C. Virion composition was confirmed by immunoblotting. The p24 content of viral stocks was determined using HIV-1 p24 Enzyme-linked Immunosorbent Assays (ELISA, NEK050; PerkinElmer, MA, USA), while infectivity of viral stocks was tested on TZM-bl cell line, all as previously published^[30]^.

For h-microglia infection with pseudotyped YU-2, NL4-3 or NL4-3 Δnef, volume of viral stock containing 500 ng of HIV-1 p24 was added to 1 × 10^7^ cells in 15 mL of complete DMEM, and incubated overnight at 37 °C. The next day, the excess virus was removed; cells were washed with DPBS and cultured for the indicated times in DMEM supplemented with EV-depleted FBS.

### Enrichment of EVs (and virions) from conditioned media

For EV enrichment, conditioned media of h-microglia cultures were collected 48 h after transfection of pNef.GFP or DOX-induced expression of genome integrated Nef.GFP. Briefly, conditioned medium was centrifuged at 1,000 × *g* for 20 min to remove cells and cellular debris. The same procedure was applied to all control samples. Further steps depended on the sample type: (i) total EV population; (ii) small EV population; and (iii) extracellular particle population.

(i) For total EV population enrichment, processed media were concentrated using Amicon Ultra-15 Centrifugal Filter Units (100 kDa; Merck Millipore, USA), diluted with DPBS to a final volume of 30 mL and ultracentrifuged at 100,000 × *g* for 1 h at 4 °C (MLA-50, Beckman Coulter (BC), USA). The crude EV pellet was resuspended in 40 µL of DPBS, supplemented with protein inhibitors, and stored at -20 °C until further analysis by TEM, structured illumination microscopy (SIM) or asymmetric flow field-flow fractionation coupled to a multi-angle light scattering detector (AF4-MALS) (for details see Sections Microscopy, TEM and AF4-MALS). For immunoblotting, the pellet was resuspended in 1 mL of DPBS and processed at 100,000 × *g* for 1 h at 4 °C (TLA-55, BC, USA). The final pellet was resuspended in radioimmunoprecipitation assay (RIPA) buffer supplemented with protease inhibitors (Sigma Aldrich, USA), and frozen at -20 °C. Alternatively, following the initial ultracentrifugation, the pellet was resuspended in total volume of 400 µL DPBS, transferred on top of the 20%-60% discontinuous sucrose gradient, and ultracentrifuged at 100,000 × *g* for 18 h at 4 °C (MLS-50, BC, USA). Twelve 400 µL fractions collected from the top of the gradient were precipitated with trichloroacetic acid (TCA), resuspended in 1 × NuPAGE^TM^ LDS sample buffer (Invitrogen, Thermo Fisher Scientific, USA) and frozen at -20 °C until further use.

(ii) For small EV population enrichment, processed media were either filtered through a 0.22 µm filter (Merck Millipore, USA) or, in the case of h-microglia stably expressing Nef.GFP (or GFP), subjected to 10,000 × *g* centrifugation for 30 min at 4 °C. For pelleted crude small EVs, the first step of ultracentrifugation was performed at 100,000 × *g* for 70 min at 4 °C (MLA-50 or 50.2 Ti; BC, USA). The pellet was resuspended in 1 mL of DPBS, followed by additional ultracentrifugation at 100,000 × *g* for 70 min at 4 °C (TLA-55, BC, USA). The final pellet was resuspended in 80 µL of DPBS and stored at -20 °C until further analysis by NTA, nano-flow cytometry and immunoblotting (for details see Sections NTA, Nano-flow cytometry analysis and Immunoblot analysis). Alternatively, the pellet from the first ultracentrifugation step was resuspended in DPBS to a total volume of 700 µL and transferred on top of discontinuous iodixanol density gradient (ODG; Optiprep, Sigma-Aldrich, USA), prepared by layering 2.9 mL of 40%, 20%, 10%, and 2.5 mL of 5% iodixanol solutions from bottom to top (as described in the work by Geeurickx *et al*.^[35]^). The gradient was ultracentrifuged at 100,000 × *g* for 18 h at 4 °C (SW40, BC, USA) without brake. Next, pelleted EVs or twelve 1 ml ODG fractions were collected and directly analyzed by NTA, nano-flow cytometry, and immunoblotting. For immunogold TEM and nano-flow cytometry, fractions 5-9 (1.097-1.196 g/mL) were pooled, concentrated with Amicon Ultra-15 Centrifugal Filter Units (100 kDa; Merck Millipore, USA) and stored at -20 °C until further use.

(iii) For enrichment of extracellular particles in the culture media of h-microglia infected by VSV-G HIV-1, conditioned media were filtered through a 0.22 µm filter (Merck Millipore, USA). The supernatant was carefully layered over 2 mL of 20% sucrose (Merck Millipore, USA) in DPBS and subjected to ultracentrifugation at 100,000 × *g* for 2 h at 4 °C (SW-28 rotor, BC, USA). Pellets containing EVs and/or virions were resuspended in 500 μL of DPBS and separated on 6%-18% ODG (Optiprep, Sigma Aldrich, USA) at 250,000 × *g* for 2 h, at 4 °C (SW-41 Ti, BC, USA). Eleven fractions were collected and analyzed with acetylcholinesterase (AChE) activity assay, HIV-1 p24 ELISA (PerkinElmer, USA), and immunoblotting after TCA and sodium deoxycholate protein precipitation (for details see Sections Immunoblot analysis, AChE activity and HIV-1 p24 capture ELISA).

### Microscopy

#### Fluorescence microscopy

h-microglia stably expressing Nef.GFP under a DOX-inducible promoter were tested for GFP expression using fluorescence microscopy. Cells were seeded at 5 × 10^4^ cells per well in Millicell EZ Slides (C86024, Merck Millicell, USA) overnight to obtain 60%-70% confluence. Next, they were washed with DPBS and the medium was changed for the EV-depleted DMEM, supplemented with (50 ng/mL) or without (control) DOX and cultured for 48 h. Post-treatment, cells were washed and fixed with 4% paraformaldehyde (PFA) solution in PBS, mounted with ProLong® Gold antifade reagent with 4’,6-diamidino-2-phenylindole (DAPI, Thermo Fisher Scientific, USA), and observed with a fluorescence microscope (Axio Imager M2, Zeiss, Germany). Images were processed using ImageJ (FIJI, Fiji Is Just ImageJ) software (National Institute of Health, Bethesda, MD, USA, available at https://imagej.net/ij/).

#### Super-resolution microscopy

Small volumes (~3 µL) of EVs in DPBS, enriched for Nef.GFP from Nef.GFP-expressing h-microglia cultures, were transferred to the poly-L-lysine-coated coverslips, sealed to the objective glass and imaged with an oil-immersion plan apochromatic differential interference contrast objective (63×/NA 1.4) using a super-resolution SIM (ELYRA PS.1, Zeiss, Germany). Fluorescent images were acquired with an electron-multiplying charge-coupled (EMCCD) camera (andor iXon 885, Andor Technology, Belfast, UK) using five grating frequencies for SIM. Nef.GFP was excited with the 488 nm diode-pumped solid-state laser line, and emission fluorescence was filtered with a bandpass filter of 495-575 nm. To estimate the apparent EV size (spot area), super-resolution SIM images were analyzed with ImageJ software. The EV diameter (2r) was calculated using r = (S/π)^0.5^. The 2r data were fitted by the Weibull function (black line): f = a*((c-1)/c)^((1-c)/c)*(abs((x-x_0_)/b+((c-1)/c)^(1/c))^(c-1))*exp(-abs((x-x_0_)/b+((c1)/c)^(1/c))^c+ (c-1)/c)). Each Nef-GFP-positive spot was extracted from the image using the 25 arbitrary units intensity threshold. The minimum spot taken to identify Nef.GFP-positive EV was five adjacent pixels (40 nm × 40 nm), and the minimum surface area (S) covered by the spot was 8,000 nm^2^; thus, a broad span of spots with different sizes was analyzed. In SIM images, either no circularity [Cir = 4π × (area)/(perimeter)^2^] was considered (Cir; 0-1), or only spots with nearly ideal circular shape (Cir, 0.99-1) were included in the analysis to calculate the spot diameter (2r). This parameter was displayed as a frequency histogram using SigmaPlot 11.0 (Systat Software Inc., USA).

### TEM

#### Negative staining

For whole-mount analysis, EVs were visualized by TEM using the negative staining method. First, 4 μL of EVs suspension was loaded onto a glow-discharged formvar/carbon-coated copper grids. Adsorbed vesicles were washed in distilled water, stained with 1% (w/v) aqueous uranyl acetate, washed in distilled water and air-dried. Samples were examined with a Philips CM 100 transmission electron microscope (The Netherlands), operating at 80 kV. Images were recorded with an Advanced Microscopy Techniques (AMT) camera (Advanced Microscopy Techniques Corp., Woburn, MA, USA).

#### Immunogold labelling

To determine the topology (luminal *vs*. surface) of EV-associated Nef.GFP, we combined the negative staining method for TEM with immunolabeling. The input sample was Nef.GFP EVs pooled from fractions 5-9 collected after ODG separation. All steps were performed at room temperature. EV samples were applied on freshly glow discharged copper grids for 3 min and fixed with 4% PFA dissolved in 0.1M phosphate buffer (PB) for 10 min. After three washing steps with 0.1% bovine serum albumin (BSA) in PB, grids with applied Nef.GFP EVs were divided: one was treated (perforated) with 0.05% Triton X-100 dissolved in PB for 4 min, while the other was incubated only with PB (nontreated EVs). The grids were washed three times prior to blocking with 1% BSA (Sigma-Aldrich, USA) in PB for 1 h. For immunolabeling, the grids were incubated on a drop of rabbit polyclonal antibodies against Nef (ab63918, Abcam, UK) (500x dilution, 1h), followed by three consecutive washing steps and incubation of the grids on a drop of goat-anti-rabbit immunoglobulin G (IgG, according to antibody producer recommendation), conjugated with gold particles with 10 nm in diameter (810.011-05, Aurion, NL) (5x dilution, 20 min). After washing, fixation with 1.5% glutaraldehyde in PB, and washing with PB and miliQ water, the grids were stained with 1% (w/v) water solution of uranyl-acetate and air dried. Negative controls of both EV samples were prepared by omitting addition of antibodies against Nef. Nef.GFP EVs were also analyzed by the negative staining method only (without immunolabeling). The grids were observed using a TALOS L120C transmission electron microscope (Thermo Fisher Scientific, USA) operated at 100 kV, and representative micrographs (camera Ceta 16 M) were taken using Velox software (Thermo Fisher Scientific, USA).

### Asymmetric-flow field-flow fractionation coupled to a multi-angle light-scattering detector (AF4-MALS)

AF4 was performed at room temperature on an Eclipse 3+ system (Wyatt Technology Europe, Germany) connected to an isocratic pump, an online vacuum degasser and an autosampler (all Agilent Technologies 1260 series, USA). Samples were separated in a channel with a trapezoidal spacer with a thickness of 350 µm, a tip-to-tip length of 152 mm and an initial channel width of 21 mm that decreased to 3 mm. A 10 kDa regenerated cellulose membrane was used at the accumulation wall. The fractionated particles were detected with an ultraviolet (UV) detector at 280 nm (Agilent Technologies, USA), and a multi-angle light-scattering (MALS) detector (DAWN HELEOS, Wyatt Technology, USA) operated at 658 nm, calibrated with toluene and normalized with BSA protein as an isotropic scatterer. Phosphate-buffered saline (PBS, pH 7.4) was used as a running eluent and consisted of 137 mM NaCl, 2.68 mM KCl, 10.14 mM Na_2_HPO_4_, and 1.84 mM KH_2_PO_4_. It was supplemented with 0.02% w/v sodium azide (NaN_3_) as bactericide and filtered through a Nylon 66 membrane with a pore size of 0.45 µm (Supelco Analytical, USA). An additional inline filter with a pore size of 0.1 µm was inserted between the HPLC pump and the AF4 channel.

Typically, 30 µL of the EV samples were injected in focus mode with a focus flow of 1.5 mL/min and an injection flow of 0.2 mL/min over 5 min. After injection, the samples were focused for an additional 7 min. After focusing, the samples were eluted at a detector flow rate of 1.0 mL/min using two successive, linear cross-flow gradients, i.e., 3-0.25 mL/min in 10 min and 0.25-0.09 mL/min in 45 min. In all experiments, the last two steps, i.e., elution and elution + injection, included washing of the channel and injection loop without cross-flow. Astra 5.3.4.20 software was used for data acquisition and evaluation. The size of the EVs was expressed by the root mean square radius (*R*_rms_) obtained from the MALS detector. The *R*_rms_ values of the fractionated EVs were calculated using data from 15 angles of the MALS detector. The amount of eluting EVs was evaluated from MALS data using the Astra number density template and the EV refractive index of 1.39^[36]^.

### Nanoparticle tracking analysis (NTA)

Particle concentration and size in EV-enriched samples were determined by NTA using the NanoSight NS300 instrument (488 nm laser) connected to an automated sample assistant (both Malvern Panalytical, UK) with constant syringe pump flow. Dilutions of 10- or 200-fold in particle-free DPBS were used. Five recordings of 60 s were performed and captured at camera level 14. After visual inspection of all recordings, the raw data was analyzed using the NanoSight NTA 3.3 software at the following settings: detection threshold 5, sample viscosity as the corresponding viscosity for water, temperature 25 °C, automatic settings for minimum expected particle size and blur, and minimum track length 10. Only data with a minimum of ten particles per frame (PPF) corresponding to ≥ 1,500 individual particle tracks were included in calculations. The output data was calculated as total particle number (particle No.) per 10^6^ cells and particle size (mode hydrodynamic diameter in nm). The results were analyzed using GraphPad Prism 10 (GraphPad Software, USA).

### Nano-flow cytometry analysis

The Flow NanoAnalyzer U30E (NanoFCM, UK) was used to measure the concentration of all particles and Nef.GFP (or GFP) positive particles following the manufacturer’s instructions. Briefly, single-photon counting modules were used to simultaneously detect the side scatter (SSC) (using a bandpass filter 488/10 nm) and green fluorescence (bandpass filter 525/40 nm) of individual particles. The instrument was calibrated for particle concentration using 250 nm standard fluorescent silica beads [Quality Control (QC) beads] and for particle sizing using pre-mixed silica beads with diameters of 68, 91, 113 and 155 nm (S16M-Exo beads). Individual (and pooled) fractions after EV separation on the density gradient were diluted 100- or 200-fold in Tris-EDTA (TE) buffer (pH 7.5) to reach a particle count within the optimal range of 2,000-12,000 during a 1-min measurement at a pressure of 1 kPa. Analysis was conducted using NanoFCM Profession 3.0 Software (NanoFCM, UK). The raw data were analyzed using GraphPad Prism 10 (GraphPad Software, USA).

### Immunoblot analysis

h-microglia cells were lysed in RIPA lysis buffer [1% IGEPAL CA-630 (Sigma-Aldrich, USA), 0.1% sodium dodecyl sulfate (SDS) and 0.5% sodium deoxycholate in DPBS], supplemented with protease inhibitors (Protease Inhibitor Cocktail; I3911-1BO; Sigma-Aldrich, USA) for 15 min at 4 °C and then centrifuged at 12,000 × *g* for 15 min at 4 °C. The supernatant was collected and stored at -20 °C until further use. The total amount of cell lysate (CL) and EV proteins was estimated with the Pierce Bicinchoninic Acid (BCA) Protein Assay Kit (Thermo Scientific, USA). Additionally, 25-30 µg of CL, total EV proteins or proteins from TCA-precipitated EV fractions were separated by 4%-12% Bis-Tris SDS polyacrylamide gel electrophoresis (SDS-PAGE), (NuPAGE, Thermo Fisher Scientific, USA) and transferred to the poly(vinylidene fluoride) (PVDF) membrane (Merck Millipore, USA).

Primary antibodies used were as follows: mouse monoclonal antibodies against AChE (MAB303, Millipore, USA), Alix (2171, Cell Signaling Technology, USA), CD81 (NBP1-44861, Novus Biologicals; USA), Cytochrome C (556433, BD Biosciences, USA), Flotillin (610820, BD Biosciences, USA), GAPDH (G8795, Sigma-Aldrich, USA), GFP (sc-9996, Santa Cruz Biotechnology, USA), HIV-1 Nef (ab42355, UK), Hsp70 (ab5442, Abcam, UK), p24 (ab9071, Abcam, UK) and Tsg101 [4A10] (ab83, Abcam, UK); or goat polyclonal antibodies against Actin (sc-1615, Santa Cruz Biotechnology, USA), Annexin A2 (sc-1924) and HSC70 (sc-1059); or rabbit polyclonal antibodies against Calnexin (sc-11397), CD63 (sc-15363), HIV-1 gp120 (NBP1-76371, Novus Biologicals, USA) and HIV-1 Nef (2949, BEI Resources Repository, USA); and rabbit monoclonal antibodies against Albumin (ab192603, Abcam, UK) and CD9 (13403, Cell Signaling Technology, USA).

Horseradish peroxidase (HRP)-conjugated secondary antibodies (anti-mouse, anti-goat, and anti-rabbit; Jackson ImmunoResearch Laboratories, USA) were used for detection. Membranes were developed using Luminata Forte Western HRP substrate (Millipore, USA) or SuperSignal West Pico Chemiluminescent Substrate (Thermo Fisher Scientific, USA) and chemiluminescence detected on ImageQuant LAS-4000 (Fujifilm, Japan) or iBright Imaging System (Thermo Fisher Scientific, USA).

### Acetylcholinesterase (AChE) activity

AChE activity was determined as described previously^[37]^. Briefly, 100 μL of each fraction collected from the Optiprep density gradient was diluted with DPBS to 180 μL and incubated with 20 μL mixture of 1.25 mM acetylthiocholine (A5751; Sigma Aldrich, USA) and 0.1 mM 5,5’-dithiobis(2-nitrobenzoic acid) (D8130, Sigma Aldrich, USA) for 30 min at 37 °C. The absorbance was measured at 412 nm using a Synergy 2 Multi-Mode Reader spectrophotometer (BioTek Inc., Germany) and the activity was expressed as pmol/µl*min.

### HIV-1 p24 capture ELISA

The HIV-1 p24 concentration was determined in 100 μL of undiluted samples using HIV-1 p24 capture ELISA assay (NEK050; PerkinElmer, MA, USA) following the manufacturer’s instructions. Absorbance of each sample was measured at 450 nm, and HIV-1 p24 concentration (pg/mL) was calculated based on the standard curve of positive samples.

## RESULTS

### h-microglia transiently expressing Nef.GFP release EVs with distinct characteristics

**Figure 1:**
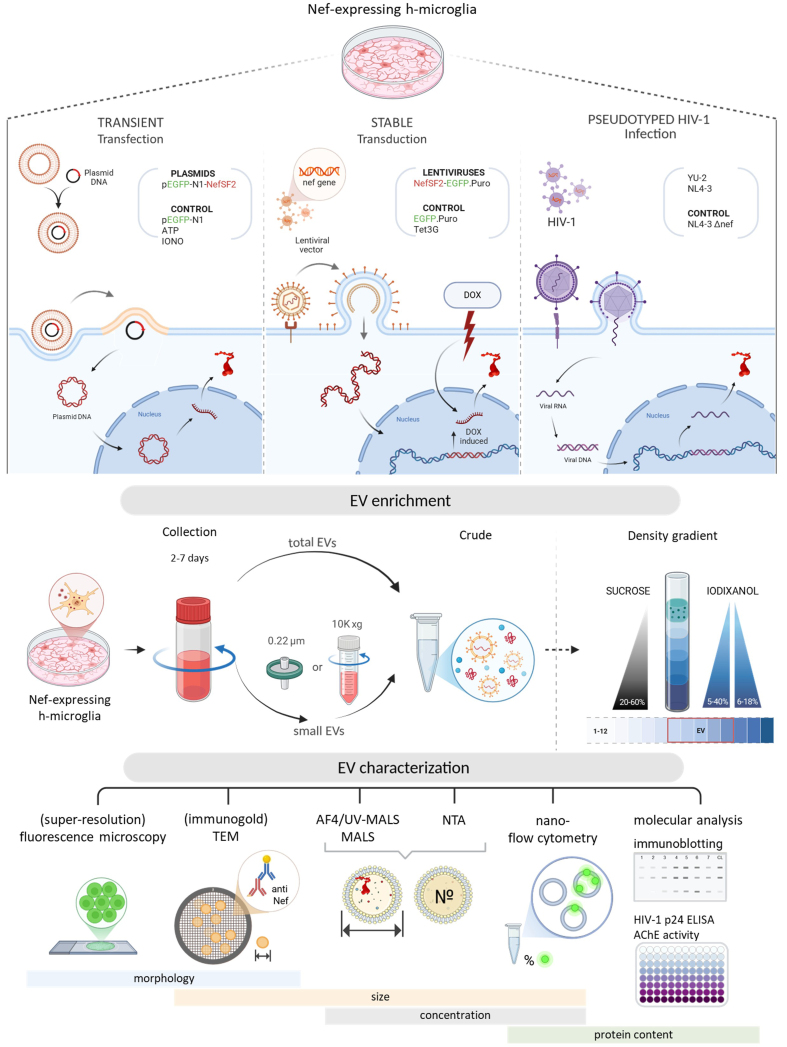
Schematic overview of the experimental workflow. Top panel: Three cellular models used to study the effect of Nef expression in h-microglia on vesiculation: transient expression following plasmid transfection, regulated expression from an integrated gene via lentiviral transduction, and expression from an integrated provirus after HIV-1 infection; Middle panel: Approaches for EV enrichment from conditioned media, including differential centrifugation and density gradient fractionation, to obtain total EVs or small EVs; Bottom panel: Techniques used to study EV characteristics; size, concentration and protein content were studied by (super-resolution) fluorescence microscopy, (immunogold) TEM, AF4-MALS, NTA, nano-flow cytometry, immunoblotting and other molecular approaches. DOX: Doxycycline; EV: extracellular vesicles; TEM: transmission electron microscopy; AF4-MALS: asymmetric flow field-flow fractionation coupled to a multi-angle light scattering detector; NTA: nanoparticle tracking analysis; HIV: human immunodeficiency virus.

Our previous work detailed Nef.GFP intracellular trafficking in transfected immortalized h-microglia and demonstrated that vesicle-like structures carrying Nef.GFP are continuously released extracellularly^[25]^. To identify and further characterize the released vesicle-like structures, we transiently expressed Nef.GFP in h-microglia, isolated extracellular particles from culture media using established EV enrichment methods and performed morphological, quantitative, and molecular analyses [Figure 1].

**Figure 2:**
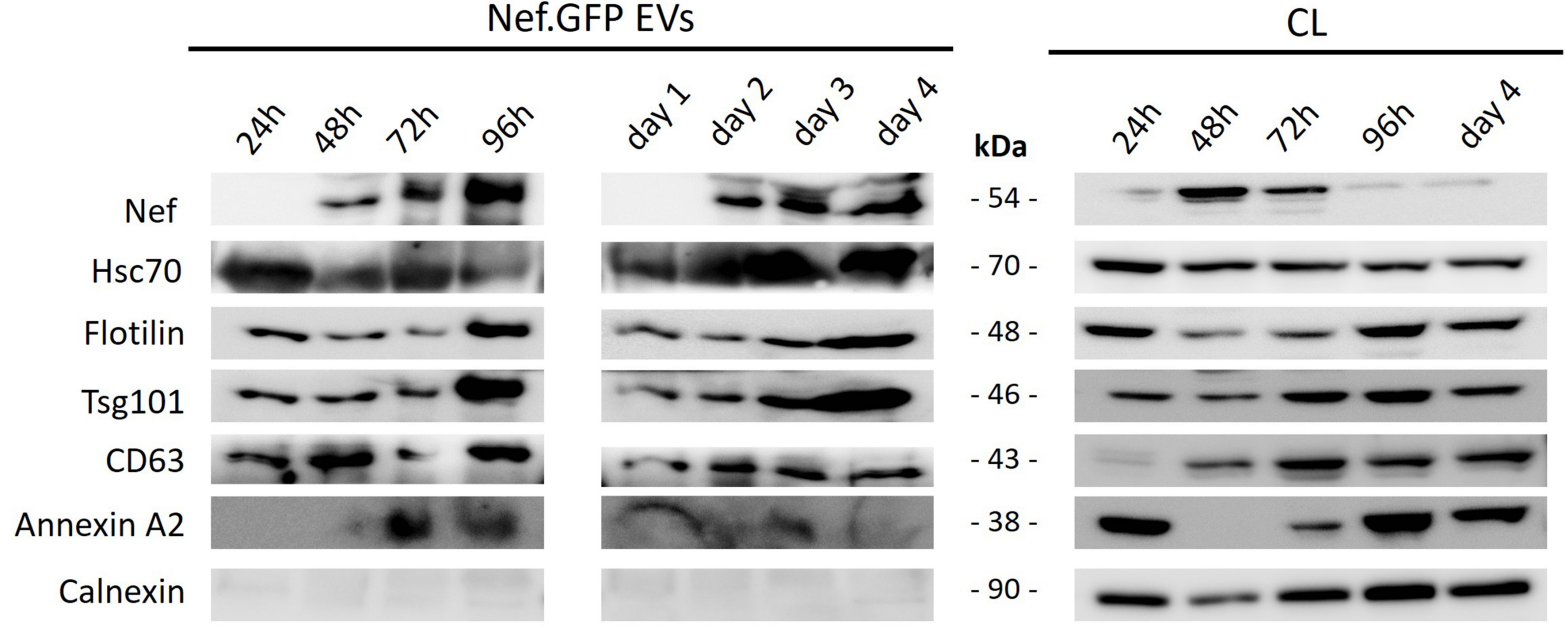
Continuous release of Nef.GFP-positive vesicle-like structures from h-microglia transiently expressing Nef.GFP. Immunoblot analysis of crude EVs enriched from the media of h-microglia cultures transiently expressing Nef.GFP at the indicated times, either cumulatively after 24, 48, 72, and 96 h in culture, or by sampling the same culture on days 1, 2, 3, and 4, with growth media replaced after each sampling. For samples, cells were collected at the end points in both growth experiments, that is after 24, 48, 72 and 96 h, and after day 4. Antibodies were directed against GFP, typical EV proteins (Hsc70, Flotillin, Tsg101, CD63, Annexin A2) and EV impurity marker Calnexin. CL: Cell lysate; Nef.GFP: Nef green fluorescent protein; h-microglia: human microglia; EV: extracellular vesicle; Hsc70: heat shock cognate 70; Tsg101: tumor susceptibility gene 101; CD63: cluster of differentiation 63; Annexin A2: annexin A2 protein.

To first address the kinetics of vesicle-like structures release, we performed time-growth experiments and collected media from h-microglia cultures expressing Nef.GFP after 24, 48, 72 or 96 h following transfection, or grew h-microglia continuously and collected media after day 1, day 2, day 3 and day 4. We processed media by simple ultracentrifugation and washing steps to enrich the total EV population, and performed immunoblotting analysis on CLs and pelleted particles [Figure 2]. We observed weak Nef.GFP signal in CLs at 24 h, reaching maximal expression at 48 h, and declining to nearly undetectable levels by 96 h. Importantly, the enriched EV samples showed a continuous release of Nef.GFP-positive vesicle-like structures from day 2 to day 4, which remained stable and progressively accumulated in h-microglia cultures between 48-96 h. Since h-microglia cultures showed high Nef.GFP expression and viability with detectable extracellular release of Nef.GFP at 48 h post-transfection, we performed all further experiments at this time-point.

**Figure 3:**
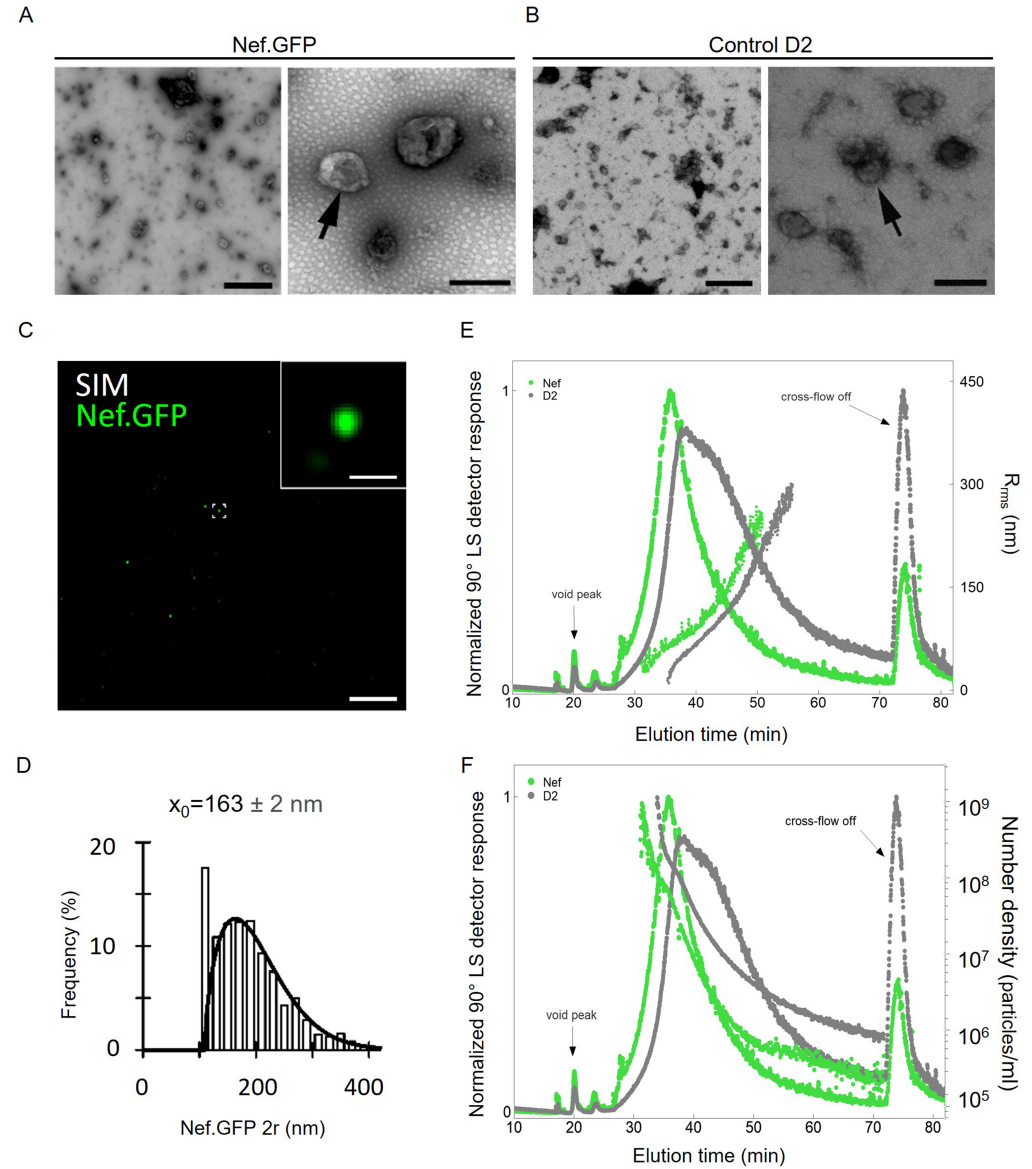
Transient expression of Nef.GFP in h-microglia stimulates extracellular release of smaller vesicles carrying Nef.GFP. Using simple ultracentrifugation, crude total EVs were enriched from media of non-transfected h-microglia cultures (control D2) or 48 h post transfection (Nef.GFP). Representative transmission electron microscopy images of negatively stained pelleted particles from the (A) Nef.GFP and (B) control D2 samples. Scale bars: 1 µm (left) and 200 nm (right). Arrows indicate single extracellular particles in higher magnification; (C) Representative SIM image of pelleted particles in the Nef.GFP sample, attached to the coverslip surface and observed in super-resolution mode; scale bar, 5 µm. One of the Nef.GFP-positive EVs (green; framed in center) is magnified in the top right corner; inset scale bar: 0.5 µm; (D) Frequency (%) plot of diameter distributions (2r, nm) for 1619 morphologically distinct Nef.GFP‑positive EVs. The mean EV 2r (x_0_ ± s.e.) is displayed above the plot; (E) Normalized AF4-MALS fractograms recorded by 90° LS detector (solid lines) for pelleted particles from the control D2 (gray) and Nef.GFP (green) samples with displayed root-mean-square radius, R_rms_ (filled circles). (F) Normalized AF4-MALS fractograms recorded by 90° LS detector (solid lines) for pelleted particles from the control D2 (gray) and Nef.GFP (green) samples, with particle number density per mL (filled circles) as a function of elution time. AF4-MALS: Asymmetric-flow field-flow fractionation coupled to a multi-angle light-scattering detector; SIM: structured illumination microscopy; Nef.GFP: Nef green fluorescent protein; h-microglia: human microglia; EV: extracellular vesicle; LS: light scattering; R_rms_: root-mean-square radius; D2: day 2 post-transfection.

The TEM analysis of negatively stained pelleted particles [Figure 3A and B] detected EVs with typical cup-shaped structures when dehydrated, in samples from both Nef.GFP-expressing (Nef.GFP) and non-transfected (control D2) h-microglia cultures. Using super-resolution fluorescence microscopy [Figure 3C and D], we found that some particles in the Nef.GFP sample contained Nef.GFP (green spots). By analyzing inverse contrast images after segmentation, and considering that a minimum particle consists of ≥ five adjacent pixels and has a variable shape, we determined the mean diameter of the Nef.GFP-positive particles as 163 ± 2 nm [with realistic lateral (xy) resolution limit of ~100-120 nm]^[38,39]^.

To further quantify total EV-enriched samples, we performed asymmetric-flow field-flow fractionation coupled with MALS detector (AF4-MALS) [Figure 3E and F]. The elution profile recorded by the 90° light scattering (LS) detector displayed one peak with an apex at elution time of 35 min for the Nef.GFP sample (green line), while the control D2 sample with peak apex at 37 min showed a somewhat broader size distribution (grey line; Figure 3E). The corresponding average R_rms_ of particles was 172 nm for the Nef.GFP sample and 282 nm for the control D2 sample [Supplementary Table 1]. In comparison, adenosine triphosphate (ATP) and ionomycin, known stimulants of EV secretion from microglia^[40]^, induced the release of larger particles from h-microglia, as indicated by TEM [Supplementary Figure 1A] and measured by AF4-MALS (R_rms_ 340 nm and 422 nm, respectively, Supplementary Table 1 and Supplementary Figure 1B). Additionally, based on the AF4-MALS data, h-microglia expressing Nef.GFP released 11.7-fold more particles than control D2 (73.6 × 10^7^ *vs*. 6.27 × 10^7^ particles per million cells; Figure 3F and Supplementary Table 1), the latter releasing similar levels as h-microglia stimulated by ATP or ionomycin [Supplementary Table 1 and Supplementary Figure 1C]. Altogether, transient expression of Nef.GFP in h-microglia stimulated release of smaller extracellular particles, many EVs in nature, which also contained Nef.GFP.

**Figure 4:**
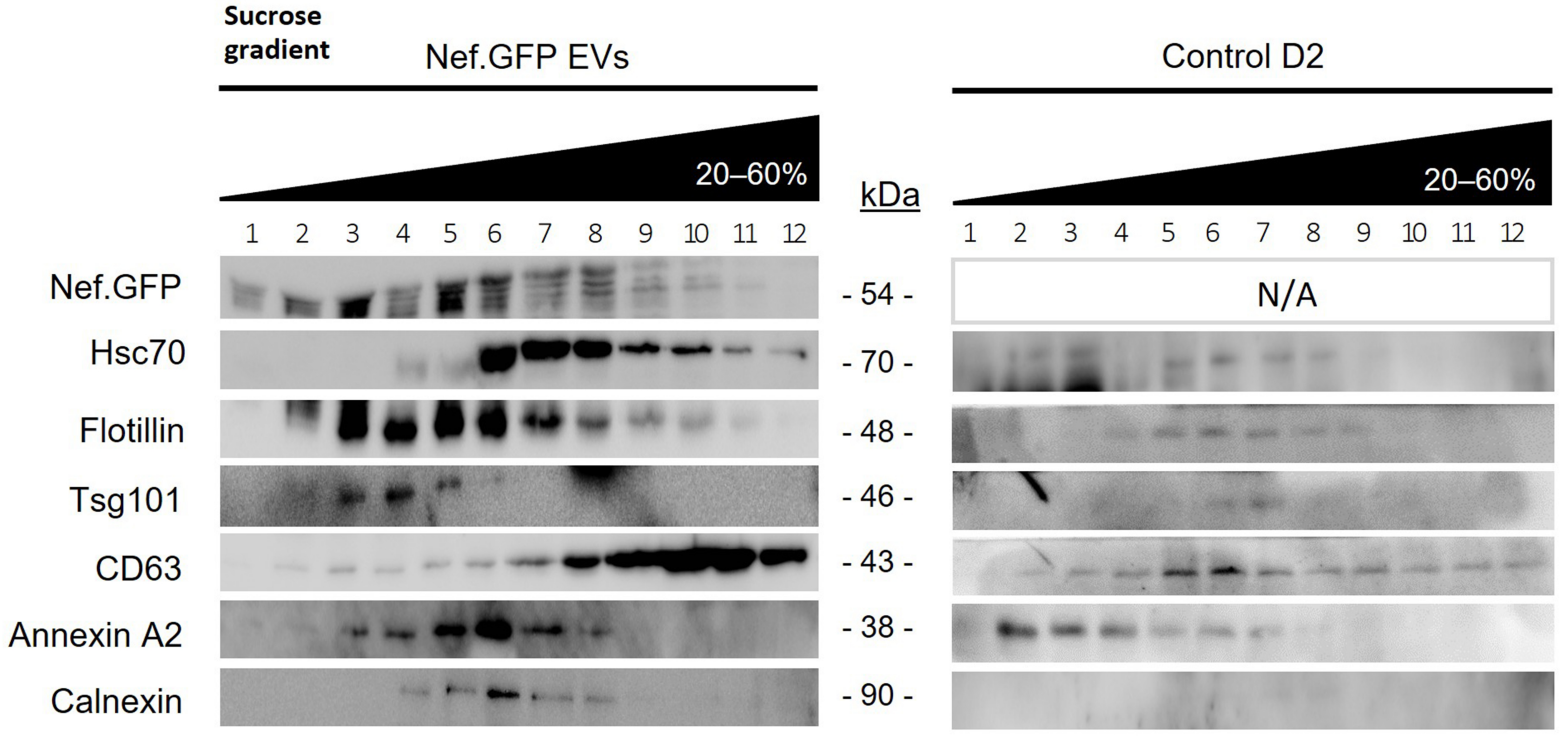
Transient expression of Nef.GFP in h-microglia affects protein composition and buoyant density of extracellular vesicles. Total EVs were enriched by simple ultracentrifugation from media of 48 h old, transfected h-microglia cultures, either expressing Nef.GFP or not (control D2). Pelleted particles from both conditions were separated on a 20%-60% sucrose gradient, and precipitated fractions were analyzed by immunoblotting with antibodies directed against GFP, typical EV proteins (Hsc70, Flotillin, Tsg101, CD63, Annexin A2) and EV impurity marker Calnexin. CL: Cell lysate; EV: extracellular vesicles; Nef.GFP: Nef green fluorescent protein; h-microglia: human microglia; D2: day 2 post-transfection; Hsc70: heat shock cognate 70; Flotillin: flotillin protein; Tsg101: tumor susceptibility gene 101; CD63: cluster of differentiation 63; Annexin A2: annexin A2 protein; Calnexin: calnexin protein.

To evaluate the effect of transient Nef.GFP expression on the protein composition of extracellularly released h-microglia particles, Nef.GFP and control D2 total EV-enriched samples were further separated on a sucrose density gradient and analyzed for the presence of specific EV markers by immunoblotting. When separated by sucrose gradient, Nef.GFP was found predominantly in fractions 3-6, which were also positive for EV markers Flotillin, Tsg101 and Annexin A2, while Hsc70 and CD63 were enriched in denser fractions 6-12. Calnexin displayed a weak signal in fractions 4-8, likely due to protein corona or remaining contaminants [Figure 4]. Interestingly, control D2 sample displayed different patterns of EV marker separation, generally separating into lighter fractions compared to the Nef.GFP sample. In summary, transient expression of Nef.GFP in h-microglia affected the protein composition and buoyant density of extracellularly released particles. Morphological and molecular characteristics of extracellular particles from Nef.GFP-expressing h-microglia were typical of EVs; therefore, we will refer to them as Nef.GFP EVs.

### Inducible expression of stably integrated Nef.GFP gene in h-microglia is an improved cellular model to study Nef effect on vesiculation

Our experiments indicated a unique and significant response of h-microglia to Nef.GFP in connection with vesiculation but were limited by the extent of h-microglia cells that expressed Nef.GFP after transfection with pNef.GFP (up to 40%) and the constitutive nature of the gene promoter. To fully understand the effect of Nef on h-microglia vesiculation, we therefore aimed to develop a cellular model with stably integrated Nef.GFP gene under an inducible promoter. Similar approaches were already used to study immune modulation in T cells^[41]^ and effect of Nef on dendritic cells^[42]^.

To address this, we combined the third-generation lentiviral vector system with the TET-ON inducible gene expression system to stably integrate the Nef.GFP gene under a DOX-inducible promoter into the genome of h-microglia cells (LV-Nef.GFP, Figure 1). As controls, we similarly prepared h-microglia expressing only the TET-ON 3G transactivator protein (LV-control) or with an additionally integrated GFP gene under the same DOX-inducible promoter (LV-GFP). We next tested all three cell lines for the extent of transgene expression in response to 0-500 ng/mL DOX by flow cytometry measurement of green fluorescent cells and for viability with automatic cell counter after Trypan Blue staining.

**Figure 5:**
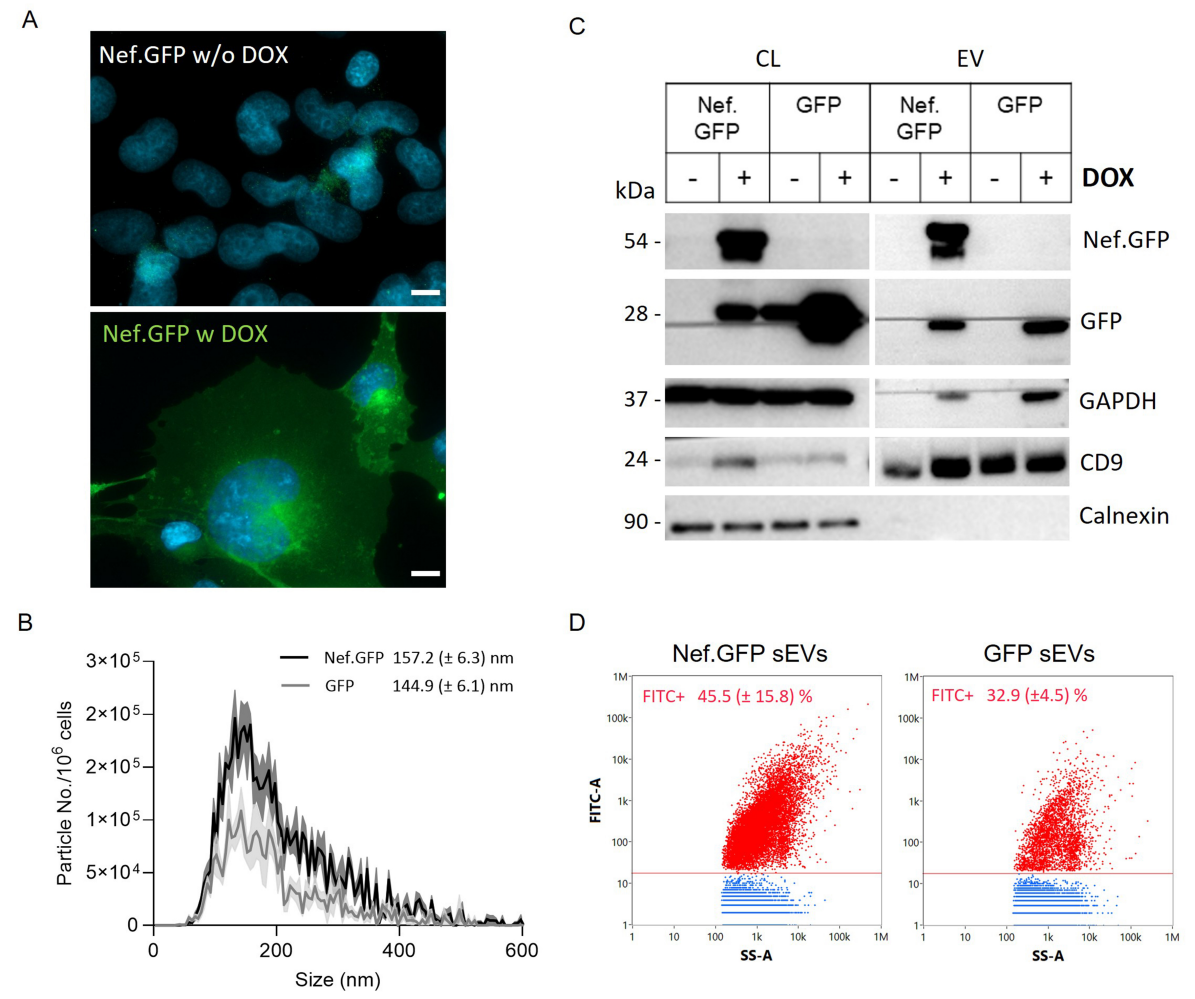
h-microglia harboring an inducible, stably integrated Nef.GFP transgene provide an improved cellular model for investigating Nef driven vesiculation. (A) Fluorescence microscopy of Nef.GFP expression in h-microglia with a stably integrated Nef.GFP transgene under an inducible promoter, without (w/o DOX) or with (w DOX) 50 ng/mL DOX treatment for 48 h. Nuclei are labelled with DAPI (blue). Scale bar: 10 μm (white); (B) Nanoparticle tracking analysis of small (crude) EVs enriched by ultracentrifugation, after removal of the 10,000 × *g* pellet from the media of Nef.GFP (black) and GFP (gray) expressing h-microglia cultures. The distribution curve represents the frequency (particles per million cells) in relation to particle mode size (2r, nm); (C) Immunoblot analysis of small (crude) EVs enriched from the media of Nef.GFP and GFP expressing (+ DOX) or not (- DOX) h-microglia cultures, with antibodies directed against Nef, GFP, typical EV proteins (GAPDH, CD9) and EV impurity marker Calnexin; (D) Representative dot plots of small (crude) EVs enriched from Nef.GFP and GFP expressing cultures, displaying FITC fluorescence (FITC-A) in relation to side scatter (SS-A) after analysis with nano-flow cytometry. The EV sample enriched from LV-control h-microglia culture was used to gate fluorescent EVs. Nef.GFP+ or GFP+ EVs are indicated in red, while non-fluorescent particles are indicated in blue. Respective percentages of fluorescent particles (FITC+) from three independent experiments (± SD) are indicated in the graph. EV: Extracellular vesicles; CL: cell lysate; Nef.GFP: Nef green fluorescent protein; h-microglia: human microglia; DOX: doxycycline; DAPI: 4’,6-diamidino-2-phenylindole; GAPDH: glyceraldehyde 3-phosphate dehydrogenase; CD9: cluster of differentiation 9; FITC: fluorescein isothiocyanate; SS-A: side scatter; LV: lentiviral; Calnexin: calnexin protein; GFP: green fluorescent protein.

LV-Nef.GFP h-microglia exposed to 50 ng/mL DOX resulted in the highest percentage of Nef.GFP-expressing h-microglia with 96.6% (± 5.0%) green fluorescent cells in the population after 48 h [Supplementary Figure 2], and high cell viability [95.4 (±4.1) %]. Fluorescence microscopy confirmed that exposure to 50 ng/mL of DOX induced Nef.GFP expression in h-microglia after 48 h in culture, while no expression was detected in the absence of DOX [Figure 5A and Supplementary Figure 2]. Live cell imaging showed that Nef.GFP protein was visibly expressed already after 6 h, with the percentage of fluorescent cells increasing over time and reaching a plateau after 36 h [Supplementary Figure 3]. LV-GFP h-microglia similarly responded well to 50 ng/mL DOX, with 98.8% (±2.3%) of green fluorescent cells in the population after 48 h [98.8% (±1.1%) viability], while no fluorescence was detected in the case of LV-control h-microglia [Supplementary Figure 2]. Altogether, h-microglia with a stably integrated Nef.GFP gene constitute an improved cellular model to study Nef effects, as they offer tightly regulated expression of Nef.GFP across the whole h-microglia population.

We next enriched small EVs from LV-Nef.GFP h-microglia culture exposed to DOX for 48 h, by including the 10,000 × *g* centrifugation step before ultracentrifugation at 100,000 × *g*, and performed NTA, immunoblotting and nano-flow cytometry analyses. NTA results supported Nef.GFP stimulated release of EVs from h-microglia, with 2.3× more particles detected in the Nef.GFP compared to GFP EV samples [Figure 5B and Table 1]. Still, the particle sizes in both Nef.GFP and GFP EV samples were similar [Table 1], which is consistent with the fact that our protocol enriched for the small EV population. Immunoblotting supported the presence of Nef.GFP in the small EV sample enriched from DOX-induced LV-Nef.GFP h-microglia, with nano-flow cytometry further determining that 45.5% (±15.8%) of all EVs contained Nef.GFP [Figure 5C and D, Table 1]. In the case of the GFP EV sample, only 32.9% (±4.5%) of EVs were positive for GFP, although expression of GFP in the cells-of-origin was higher compared to Nef.GFP [Figure 5C and D, Table 1]. Importantly, the Nef.GFP small EV sample had 4.5× more particles and 5.4× more fluorescent-positive particles compared to the GFP small EV sample [Figure 5D and Table 1]. In summary, activating the expression of a stably integrated Nef.GFP gene stimulated the general release of small EVs and the release of Nef.GFP-containing small EVs from h-microglia, in comparison to expression of the GFP gene.

**Table 1:** Characterization of particle size (in nm), number (per million cells) and fluorescence (FITC^+^) in EV-enriched samples by NTA and nano-flow cytometry

**Sample**	**EV isolation**	**NTA (mean ± SD)**		**Nano-FC (mean ± SD)**		
		**Particle No./million cells**	**Mode size** **(r in nm)**	**Particle No./million cells**	**FITC^+^ EVs/** **million cells**	**FITC^+^ (%)**
LV-control	crude	14.8 (± 12.6) × 10^7^	149.2 (± 6.6)	13.4 (± 7.57) × 10^8^	N.A.	0.2 (± 0.1)
	ODG 5-9	5.02 (± 0.71) × 10^7^	166.5 (± 14.3)	6.28 × 10^8^	N.A.	0
LV-GFP	crude	4.54 (± 2.32) × 10^7^	144.9 (± 6.1)	5.61 (± 2.48) × 10^8^	1.74 (± 0.49) × 10^8^	32.9 (± 4.5)
	ODG 5-9	2.93 (± 2.91) × 10^7^	162.0 (± 8.0)	5.13 (± 4.52) × 10^8^	0.95 (± 0.55) × 10^8^	24.3 (± 7.9)
LV-Nef.GFP	crude	10.4 (± 11.0) × 10^7^	157.2 (± 6.3)	25.0 (± 17.8) × 10^8^	9.38 (± 4.02) × 10^8^	45.5 (± 15.8)
	ODG 5-9	3.68 (± 2.57) × 10^7^	164.4 (± 11.3)	3.05 (± 1.61) × 10^8^	1.03 (± 0.43) × 10^8^	35.7 (± 3.6)

± SD were calculated from six (NTA) or four (nano-FC) independent experiments. NTA: Nanoparticle tracking analysis; nano-FC: nano-flow cytometer; r: radius; LV-control: control cell culture stably expressing inducible Tet3G; LV-GFP: cell culture stably expressing EGFP; LV-Nef.GFP: cell culture stably expressing NefSF2 with green fluorescent protein; FITC: fluorescein isothiocyanate; ODG 5-9: pooled fractions 5-9 after OptiPrep density gradient separation; N.A.: not applicable; SD: standard deviations.

### Nef.GFP is packed inside EVs released from h-microglia expressing Nef.GFP

**Figure 6:**
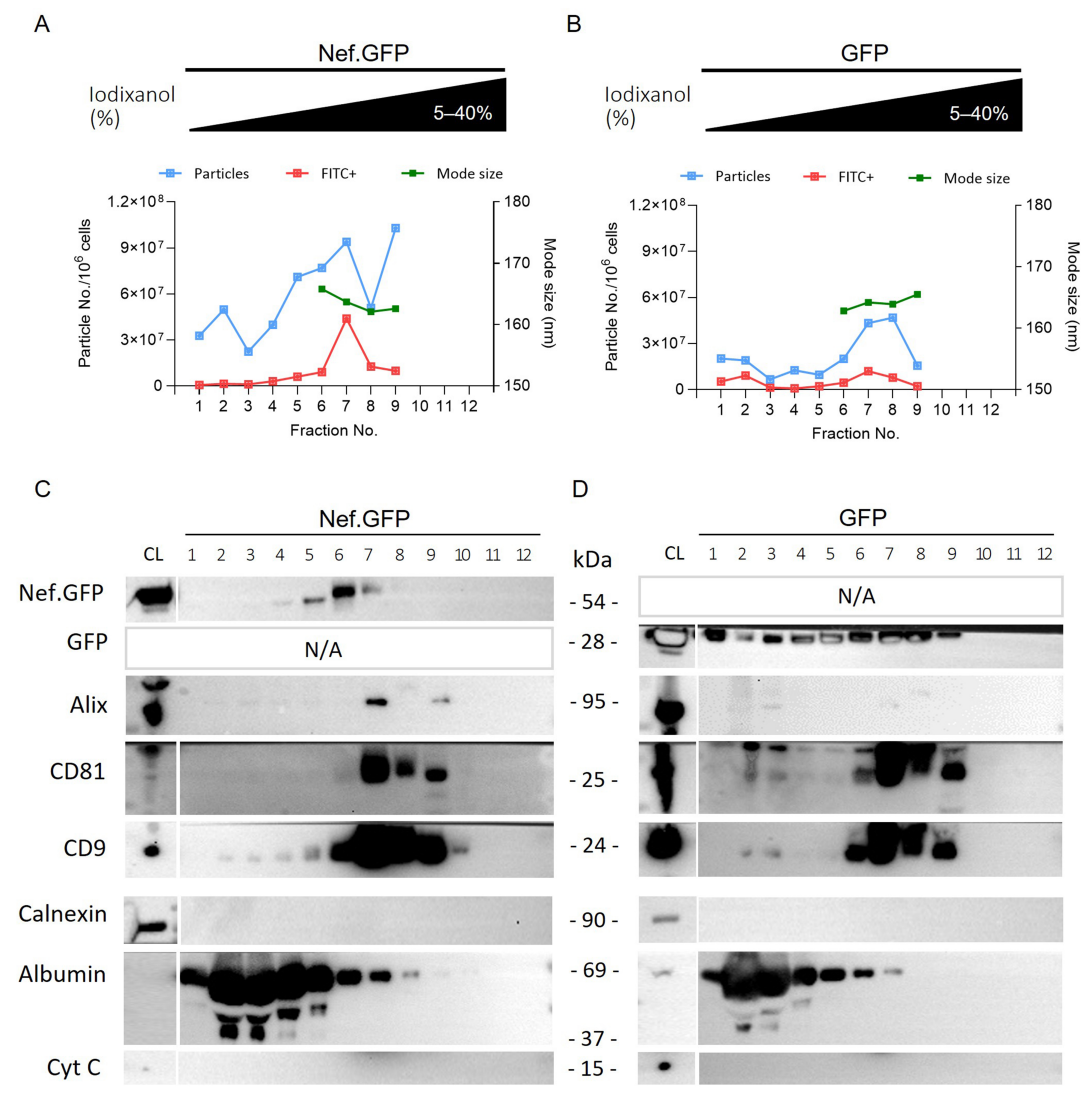
Inducible Nef.GFP expression in hmicroglia promotes the release of small Nef.GFP-positive EVs. (A and B) Crude EV samples enriched from the culture media of Nef.GFP and GFP expressing h-microglia, after removal of the 10,000 × *g* pellet, were further separated on 5%-40% iodixanol density gradient. The twelve collected fractions were analyzed by nano-flow cytometry (particles (blue) and Nef.GFP+ or GFP+ EVs (FITC^+^, red) per million cells), and nanoparticle tracking analysis [average mode size (nm; green) for fractions with at least 10 particles per frame]; (C and D) The same 12 fractions from Nef.GFP and GFP EV samples were also analyzed by immunoblotting with antibodies against GFP, typical EV proteins (Alix, CD81, and CD9), and impurity markers (Calnexin, Albumin and Cytochrome c). CL: Cell lysate; Nef.GFP: Nef green fluorescent protein; h-microglia: human microglia; GFP: green fluorescent protein; EV: extracellular vesicle; FITC: fluorescein isothiocyanate; Alix: ALG-2-interacting protein X; CD81: cluster of differentiation 81; CD9: cluster of differentiation 9; Calnexin: calnexin protein; Albumin: serum albumin; Cytochrome c: cytochrome c protein.

To further characterize Nef.GFP EVs released from the newly established h-microglia model, we performed additional separation of small EVs enriched from LV-Nef.GFP and LV-GFP h-microglia cultures exposed to DOX over the iodixanol-based density gradient (ODG; Figure 1). The twelve collected ODG fractions were next analyzed by NTA, nano-flow cytometry, and immunoblotting [Figure 6 and Supplementary Figure 4].

ODG fractions 5-9 (densities 1.097-1.196 g/mL, Supplementary Table 2) contained the highest number of particles for the Nef.GFP EV sample, with up to 46.8% of particles in each fraction containing Nef.GFP, as shown by NTA and nano-flow cytometry [Figure 6A, Supplementary Figure 4 and Supplementary Table 3]. Immunoblotting supported the presence of Nef.GFP in ODG fractions 5-7, which partly colocalized with fractions positive for Alix and CD81 (ODG 7-9), and for CD9 (ODG 5-9). Impurity markers such as albumin, calnexin, and cytochrome c were largely or completely absent from the Nef.GFP-positive fractions [Figure 6C]. Most of the particles of the GFP EV sample similarly separated into ODG fractions 5-9 as shown by NTA and nano-flow cytometry, but GFP signal was spread out over several fractions (ODG 1-9) on immunoblot [Figure 6B and D, Supplementary Figure 4 and Supplementary Table 3]. In the next step, we therefore pooled the ODG fractions 5-9 for both EV samples. Interestingly, pooled ODG fractions retained only 12.2% of particles from the crude EV sample input in the case of LV-Nef.GFP, compared to 91.4% of particles retained in the case of LV-GFP [Table 1]. The large loss of particles in the LV Nef.GFP EV sample likely reflects that many particles partitioned into the discarded ODG fractions 1-4 [Figure 6A]. Those discarded particles do not appear to carry Nef.GFP, since they are fluorescein isothiocyanate (FITC) negative [Figure 6A]. Importantly, 35.7% (±3.6%) of all EVs in the pooled sample contained Nef.GFP [Table 1 and Supplementary Figure 5].

**Figure 7:**
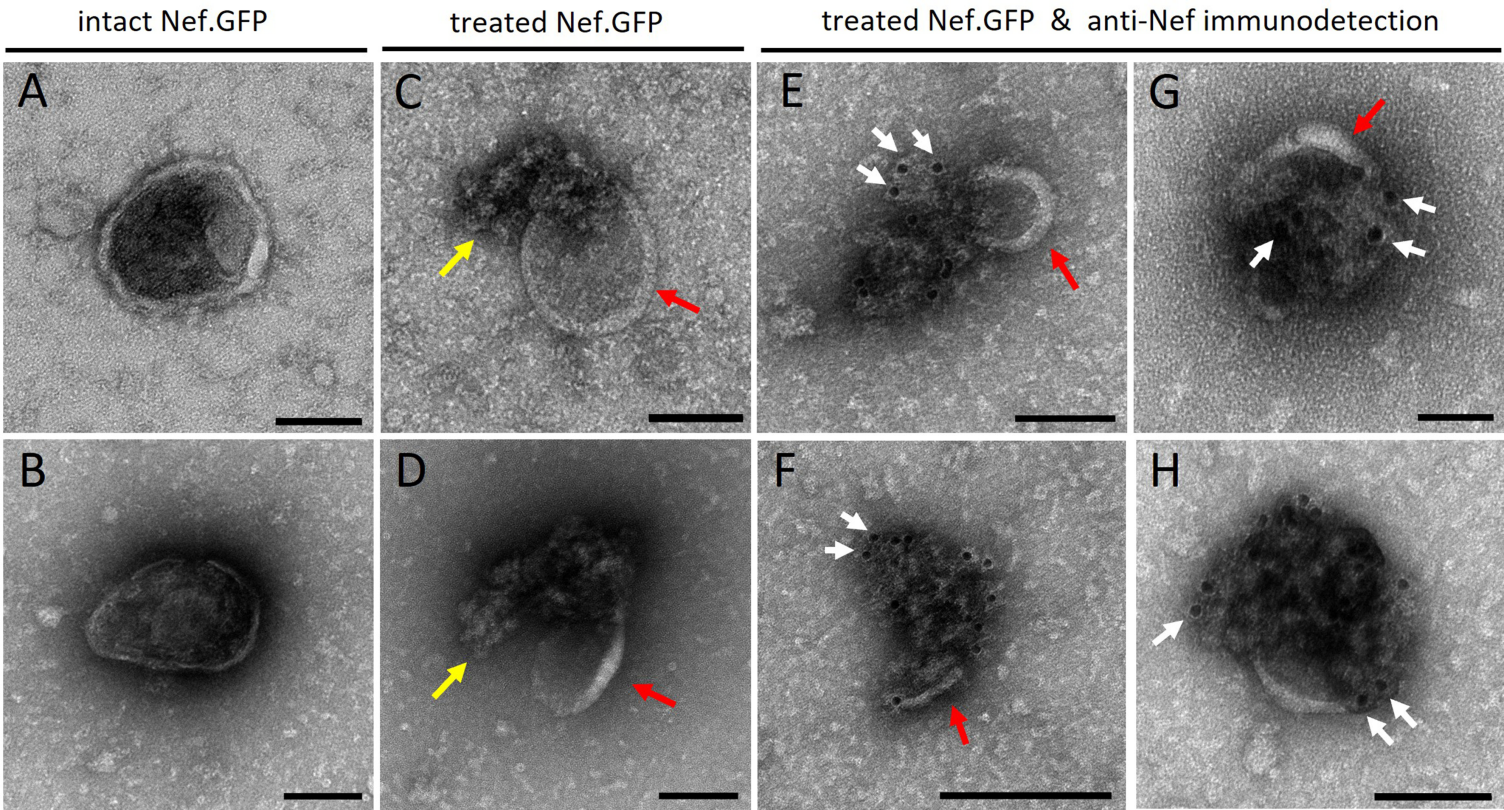
h-microglia expressing Nef.GFP release Nef sequestered inside the lumen of EVs. Transmission electron microscopy images of Nef.GFP EVs, isolated from pooled fractions 5-9 after iodixanol gradient separation of crude EVs and negatively stained with uranyl acetate. Nef.GFP EVs were either left intact or perforated with 0.05% Triton X-100 detergent, and then labeled with antibodies against Nef and IgGs conjugated to gold particles. (A and B) negative staining of intact EVs; (C and D) Negative staining of EVs treated with detergent; (E-H) Anti-Nef immunogold labelling of Nef.GFP EVs after detergent treatment. Arrows indicate membrane remnants (red), released cargo and/or damaged membrane (yellow), gold particles identifying Nef protein (white; at least three instances per micrograph). Scale bar: 100 nm (black line). To highlight individual EVs in the field of view and their potential colocalization with gold particles, images were cropped in Velox. During cropping, the software adjusts and redraws the scale bar according to the new field size, which was not uniform across the cropped images and therefore resulted in inconsistent scale bars. Nef.GFP: Nef green fluorescent protein; EV: extracellular vesicle; Triton X-100: Triton X-100 detergent; IgG: immunoglobulin G.

To understand the topology (luminal *vs*. surface) of EV-associated Nef.GFP, we performed the negative staining method for TEM combined with immunolabeling [Figure 7 and Supplementary Figure 6]. Pooled Nef.GFP EVs were mostly pure and had cup-shaped morphology, typical of dehydrated EVs. For the Nef.GFP topology analysis, pooled Nef.GFP EVs were treated with detergent Triton X-100 or left untreated, after which we performed immunogold labeling against Nef on both samples. Detergent treatment of EVs successfully perforated EVs, as we could detect released cargo [yellow arrow; Figure 7C and D *vs*. Figure 7A and B], but EVs still retained their typical structure (red arrows). Some of the released EV cargo was labeled by gold particles conjugated to IgG [white arrow; Figure 7E-H], but not all [Supplementary Figure 6F]. Gold particles were not observed in all released vesicular structures, consistent with evidence from complementary approaches indicating that Nef is present only in a subset of EVs. On the other hand, in the detergent-treated Nef.GFP EV sample, where antibodies against Nef were omitted, only a few gold particles were visible, similar to background levels, indicating the absence of nonspecific binding. By contrast, gold particles were only rarely detected in the vicinity of EVs in the case of Nef.GFP EVs not treated with detergent [Supplementary Figure 6A-D]. Altogether, this supports the conclusion that Nef.GFP is localized inside EVs.

### HIV-1-infected h-microglia release Nef into the extracellular space via EVs

Lastly, we explored Nef extracellular release from h-microglia in the context of HIV-1 infection. Microglia, as a recognized reservoir of HIV-1 in ART-treated infected individuals, could be an important source of Nef EVs in the CNS. To address this, we infected h-microglia with VSV-G-pseudotyped HIV-1 brain isolate YU-2 or the T cell-tropic molecular clone NL4-3 and analyzed cell culture media for the presence of Nef, as well as typical EV and viral proteins [Figure 1]. As controls, we additionally infected h-microglia with VSV-G-pseudotyped NL4-3 Δnef or exposed them to empty media (control). All pseudotyped HIV virions used in this study had typical protein profiles with fully processed Gag and were infective, as tested by the infectivity assay on TMZ-bl cell lines, as described previously^[30]^.

**Figure 8:**
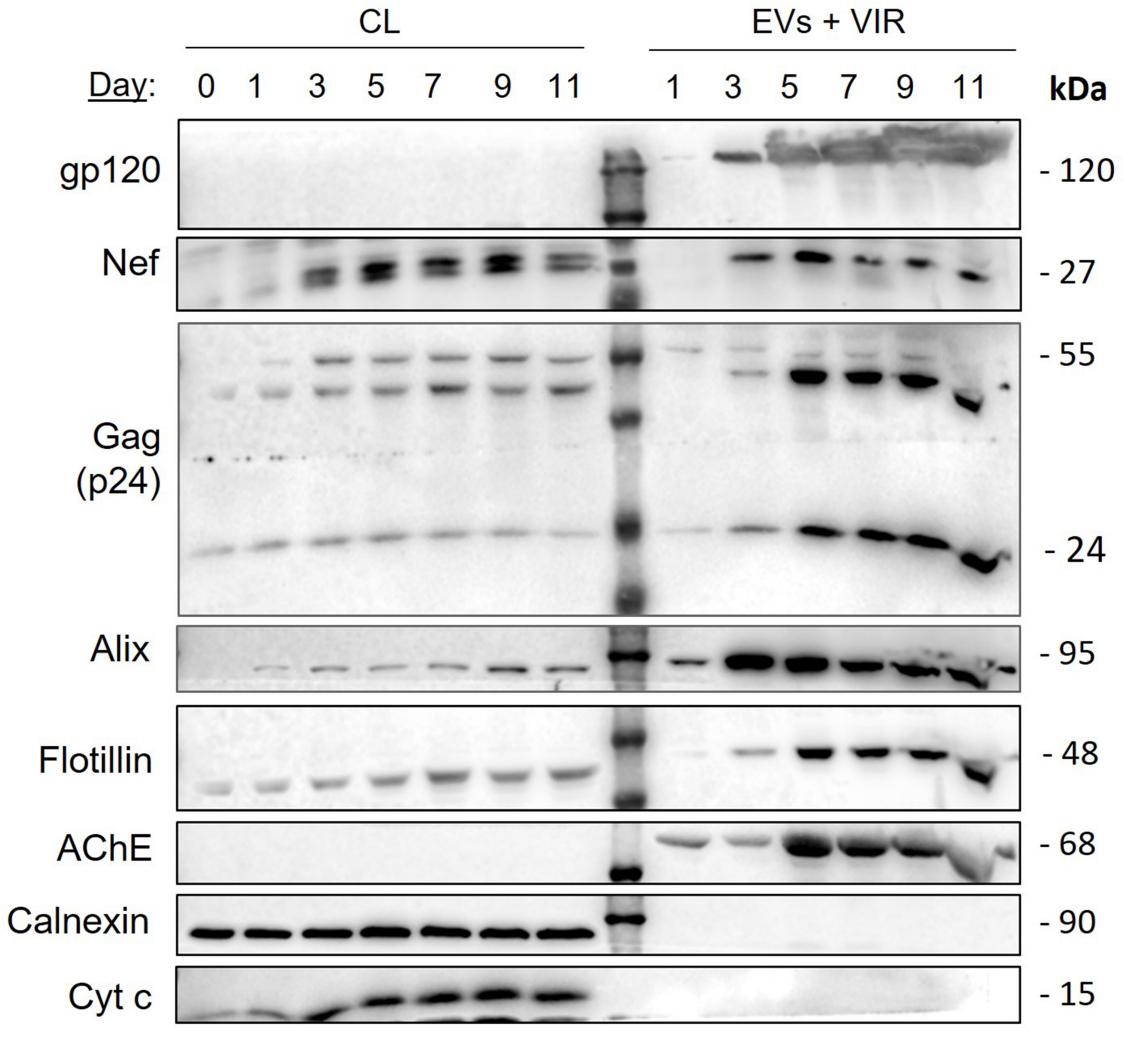
Kinetics of extracellular release of Nef from h-microglia infected with VSV-G pseudotyped HIV-1. Immunoblot analysis of cell lysates and extracellular particles (EVs + VIR) from h-microglia cultures infected with VSV-G pseudotyped NL4-3 and collected at the indicated time points. Extracellular particles were enriched from culture media by ultracentrifugation through a sucrose cushion. Antibodies directed against viral proteins (gp120, Nef, p24), typical EV proteins (Alix, Flotillin), marker of efficient gradient separation (AChE) and impurity markers (Calnexin, Albumin and Cytochrome c) were used. CL: Cell lysate; EV: extracellular vesicles; VIR: virus; AChE: acetylcholinesterase; Nef: negative factor; h-microglia: human microglia; VSV-G: vesicular stomatitis virus glycoprotein; HIV-1: human immunodeficiency virus type 1; NL4-3: HIV-1 strain NL4-3; gp120: glycoprotein 120; p24: HIV-1 core protein p24; Alix: ALG-2-interacting protein X; Flotillin: flotillin protein; Calnexin: calnexin protein; Albumin: serum albumin; Cytochrome c: cytochrome c protein.

To establish the kinetics of Nef release from HIV-1-infected h-microglia, we cultured NL4-3-infected h-microglia for 11 days, collected the culture media at the indicated time points (days 1, 3, 5, 7, 9 and 11), and processed them by ultracentrifugation through a sucrose cushion to pellet all particles, including EVs and virions. Pelleted particles and corresponding infected h-microglia CLs were then tested for the presence of Nef and selected viral and EV proteins by immunoblotting [Figure 8]. Immediately after infection, we detected low amounts of Nef in h-microglia lysates, similar to the p24 (Gag) levels, with signals increasing over time. Strong extracellular Nef signal was detected after 3 days in culture, while stronger p24 and gp120 signals appeared only after 5 days. The typical EV protein Alix showed a signal profile similar to Nef in pelleted extracellular particles, while Flotillin and AChE profiles resembled that of p24. These findings indicate that Nef is extracellularly released from HIV-1-infected h-microglia within particles distinct from virions.

**Figure 9:**
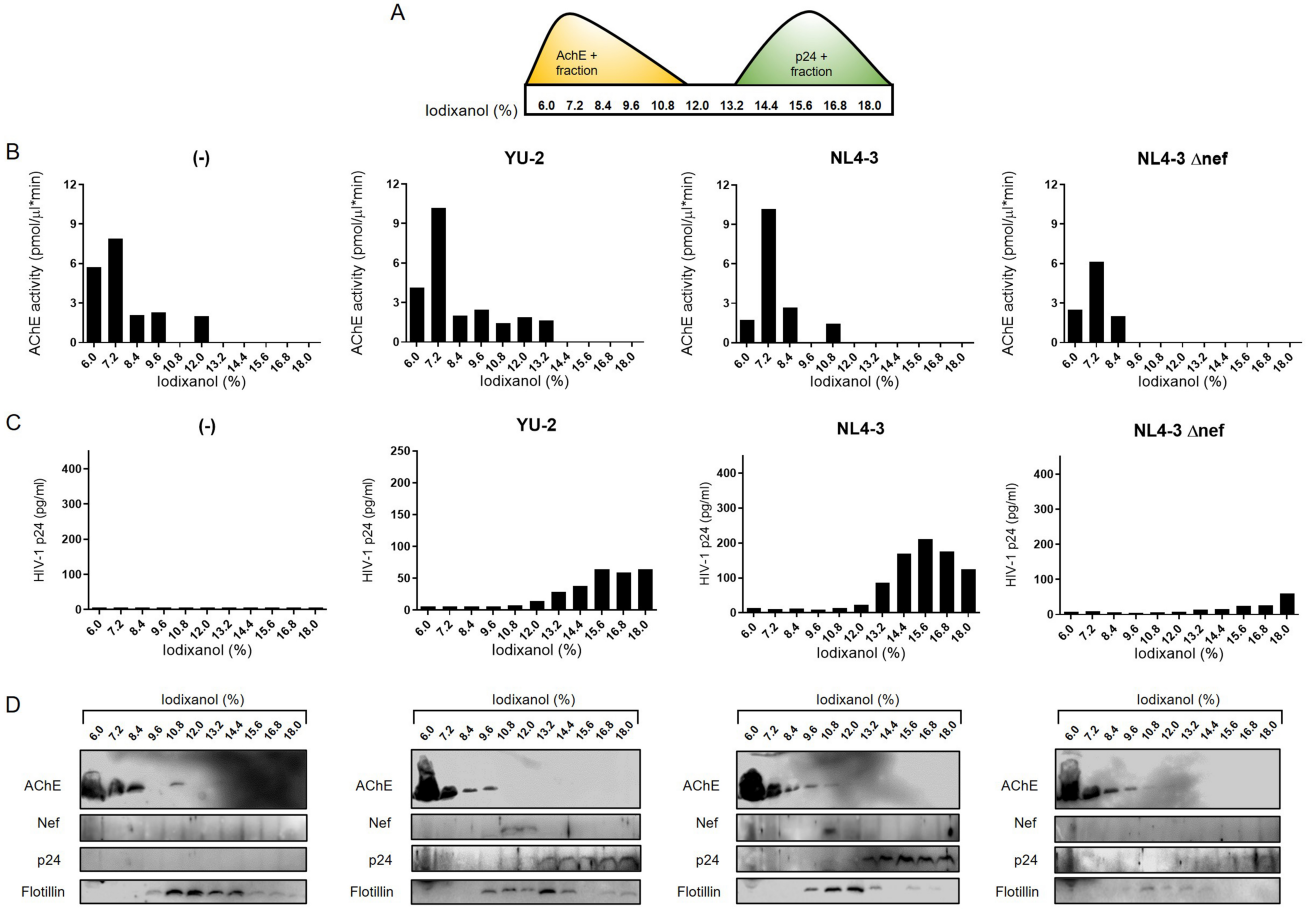
HIV-1-infected h-microglia release Nef primarily via extracellular vesicles. (A) EVs and virions pelleted from 5-day-old h-microglia cultures infected with VSV-G pseudotyped YU-2, NL4-3, NL4-3Δnef, or left uninfected (-) were further separated by 6%-18% iodixanol density gradient ultracentrifugation, and eleven fractions were collected. To support efficient separation of EVs and virions on the gradient; (B) AChE activity (y-axis) of fractions (x-axis) was quantified by colorimetric assay and (C) p24 concentration (y-axis) in fractions (x-axis) was quantified by HIV-1 p24 ELISA assay; (D) Immunoblot analysis of precipitated fractions using antibodies against AChE, Nef, p24, and Flotillin. EV: Extracellular vesicles; AChE: acetylcholinesterase; Nef: negative factor; Flotillin: flotillin protein; HIV-1: human immunodeficiency virus type 1; h-microglia: human microglia; VSV-G: vesicular stomatitis virus glycoprotein; YU-2: HIV-1 strain YU-2; NL4-3: HIV-1 strain NL4-3; NL4-3Δnef: HIV-1 NL4-3 strain with Nef gene deletion; p24: HIV-1 core protein p24; ELISA: enzyme-linked immunosorbent assay.

To further investigate virion-independent extracellular release of Nef, we collected media five days after infection of h-microglia with pseudotyped YU-2, NL4-3, or NL4-3 Δnef, or after exposure to empty media (control). We then separated EVs and virions using a previously established iodixanol gradient separation method^[28]^ and analyzed the eleven collected fractions by immunoblotting, AChE activity measurement, and p24 ELISA [Figure 9]. Although AChE is not a universal EV marker^[43]^, it can still serve as an indicator of efficient separation of virions from other particles^[28,30,44]^. AChE activity and immunoblotting signals were mainly present in lighter fractions (6.0%-12% iodixanol), whereas p24 ELISA and immunoblotting signals were detected in denser fractions (13.2%-18% iodixanol), confirming efficient separation of virions from other extracellular particles. Importantly, Nef was detected by immunoblotting in the 10.8% and 12% fractions for YU-2, and mainly in the 10.8% fraction for NL4-3 [Figure 9D]. Fractions positive for Nef signal were also positive for Flotillin but lacked p24 signal. The specificity of Nef and p24 detection was supported by the absence of Nef signal in control and NL4-3 Δnef samples, the latter still showing weak p24 signal indicative of low infection. In summary, HIV-1-infected h-microglia release Nef preferentially via EVs.

## DISCUSSION

HAND persists in many effectively treated HIV-infected individuals, partly due to brain microglial reservoirs that express low levels of viral proteins such as Nef. However, the mechanism by which microglia release Nef extracellularly, where it can exert biological functions, remains unclear. Here, using diverse h-microglia cellular models and a wide array of analytical methods, we demonstrate for the first time that Nef expression selectively induces the release of EVs carrying Nef and displaying distinct biophysical and molecular signatures. To this end, we developed a novel h-microglia model harboring a stably integrated Nef.GFP transgene under an inducible promoter. This model provides a valuable tool for studying HIV Nef biology, overcoming the major limitation of low efficiency in conventional DNA delivery methods in microglia. Using single-EV analysis techniques, we also provide the first quantification of the proportion of Nef-positive EVs (45.5% ± 15.8%) among those released from Nef.GFP-expressing h-microglia, underscoring the heterogeneity of EVs even when released from the same cell.

Expression of the HIV protein Nef fused to GFP in h-microglia, either transiently from a plasmid or stably via an inducible transgene, enhanced EV release up to 12-fold compared with h-microglia stimulated by ATP or ionomycin. This increase was largely due to elevated release of smaller EVs (R_rms_ 172 nm in case of Nef.GFP *vs*. 340 nm and 422 nm for ATP and ionomycin, respectively), which were enriched in Flotillin, Tsg101, Annexin A2 and Nef.GFP, and displayed distinct buoyant density. The divergent response to Nef.GFP *vs*. ATP and ionomycin likely reflects differences in EV biogenesis pathways. Our previous study^[25]^ identified CD9- and CD81-positive plasma membrane-derived compartments, distinct from endo-lysosomes, as sites of Nef EV biogenesis in microglia. Nevertheless, Nef.GFP expression also impaired trafficking of acidified endo-lysosomes and altered the exocytic response to Ca^2+^ levels. Rather than stimulating Ca^2+^-dependent exocytosis, ionomycin reduced mobility of dextran-laden vesicles and inhibited extracellular release of Nef.GFP. However, in the absence of Nef.GFP, ATP or ionomycin treatment of h-microglia enhanced release of large EVs, consistent with previous reports^[40,45]^. ATP activates P2X7 purinergic receptors, triggering large EV shedding through p38 MAP kinase activation, local cytoskeleton disassembly and translocation of acid sphingomyelinase to the plasma membrane, causing ceramide generation and membrane blebbing^[40,46,47]^. The effect of ionomycin has mostly been studied in the context of Ca^2+^-dependent exocytosis of multivesicular bodies and increased exosome release^[45,48,49]^. The cellular compartments involved in Nef-induced EV biogenesis may therefore be cell-type specific, since in contrast to microglia, in T lymphocytes both multivesicular bodies and the plasma membrane have been identified as sites of Nef-induced EV biogenesis^[28,50,51]^.

In h-microglia stably expressing Nef.GFP, induction stimulated the release of at least two distinct small EV subpopulations. Approximately half (45.5% ± 15.8%) carried detectable Nef.GFP, while the remainder were Nef.GFP-negative, as shown by nano-flow cytometry, immunogold TEM, and super-resolution microscopy. Notably, almost all microglial cells expressed Nef.GFP after 24 h and maintained expression through 48 h in culture. With further advances in flow cytometry-based EV sorting^[52,53]^, the specific composition and function of distinct Nef.GFP EV subpopulations can be investigated. Immunogold TEM confirmed that Nef.GFP resides within the EV lumen, as labeling was only detectable following detergent permeabilization of EVs and cargo release. This is consistent with observations that Nef localizes to the inner leaflet of the plasma membrane through N-terminal bound myristate and basic amino acids^[54-56]^, and associates with the core in intact HIV-1 virions or retroviruses formed from widely divergent Gag polyproteins^[54,57,58]^. In our study, gold particles were very rarely detected in the vicinity of EVs when the latter were not treated with detergent, indicating the absence of Nef.GFP from the EV surface. This contrasts with a recent study by Vanpouille *et al*., which reported that Nef is mostly bound to the surface of EVs from HEK293 cells^[59]^. The differing observations may be explained by differences in experimental design: (i) isolation of Nef EVs from older cultures (72 h *vs*. 48 h in our study) with higher cell death; (ii) low transfection efficiency with only 10% of cells expressing Nef (vs. nearly all cells in our study) and (iii) freezing of culture supernatants before EV isolation, which could introduce artefacts. Importantly, our observations are among the first to use single-EV analysis techniques to study the association of Nef with EVs (or other nanoparticles), whereas previous conclusions relied primarily on bulk techniques such as immunoblotting and ELISA.

Transfection of h-microglia with pNef.GFP enabled expression of Nef.GFP in only ~40% of cells after 48 h in culture, with the DNA delivery method itself potentially causing EV cargo transfer and EV functional artefacts^[60]^. EVs released from non-transfected h-microglia in such mixed culture may mask the true characteristics and functions of Nef-positive EVs within the EV population. To address this, we established a new h-microglia model harboring a stably integrated Nef.GFP transgene under an inducible promoter, which enabled a more homogenous culture in which all cells expressed Nef.GFP when exposed to DOX, thereby facilitating characterization of released Nef EVs. Similar approaches have been used to study Nef-modulated membrane trafficking and other Nef functions in T lymphocytes. For example, a tetracycline-inducible system for Nef expression was employed in Jurkat T cells to investigate the effects of Nef on signaling pathways and its association with lipid rafts during T cell activation^[61,62]^. In another model, stable expression of Nef-ER fusion protein under control of a 4-hydroxytamoxifen activator allowed investigation of temporal modulation of membrane receptors in SupT1 cells^[41]^ and the secretory activity of Jurkat cells^[27]^. Among the CNS cells, stable (but not inducible) expression of wild-type and mutant Nef in human astrocytes has been used to follow Nef-induced changes to cellular properties^[63]^. Thus, our newly developed h-microglia model may represent an important tool for studying HIV Nef biology in the context of HAND, as it is of human origin, overcomes the major limitation of low DNA delivery efficiency in microglia, and enables regulated Nef expression.

Importantly, here we show that h-microglia infected with HIV-1 isolate YU-2 or molecular clone NL4-3 preferentially release Nef by EVs, which could contribute to the pool of Nef EVs in the CNS of effectively ART-treated infected individuals. Nef release through EVs in the context of HIV infection appears to be a conserved process, as similar findings were observed in our previous studies on HIV-infected human astrocytes^[30]^ and primary T cells^[28]^, and also reported by others in Jurkat T cells infected with NL4-3 or in macaques with SIV^[29,64]^. Early studies on HIV estimated that 5-70 molecules of Nef are incorporated per virion, but since EVs and virions co-isolate due to similar physical characteristics unless separated on an iodixanol velocity gradient (as in our study)^[28,44]^, part of the detected Nef may in fact originate from EVs. Interestingly, the CD9- and CD81-positive plasma membrane-derived compartments involved in Nef EV biogenesis in h-microglia^[25]^ are also likely engaged in virion assembly upon HIV infection. Such compartments are known to develop in monocyte-derived macrophages after HIV infection and have been identified as sites of virus assembly and accumulation^[65,66]^. This is supported by previous findings that HIV-1 and EVs share biogenesis pathways^[67]^. Some studies even suggest that HIV-infected cells release a continuum of particles, ranging from classical EVs carrying viral proteins (e.g., nonreplicating particles) to fully replication-competent virions^[67]^. Experimental evidence has proposed many roles for Nef EVs in promoting chronic inflammation in the CNS by inducing oxidative stress, compromising blood-brain barrier integrity, disrupting cholesterol efflux, and enhancing lipid raft formation, which in animal models leads to reduced myelin and neuronal integrity and impairment of cognitive function^[15,16,19,26,31-33]^. Further studies are needed to clarify the contribution of Nef EVs within the broader spectrum of nonreplicating and infectious particles released from microglial HIV reservoirs in the brain.

Several limitations should be noted. First, although studies of Nef extracellular release would ideally be conducted in primary h-microglia, such investigations are limited by the scarcity of surgical brain tissue, the low abundance of microglia (approximately 10% of total brain cells), and their poor survival in culture. The h-microglia used here, although immortalized, are derived from fresh human cortical tissue and are broadly recognized as a robust model for investigating HIV latency-related processes^[34,68]^. A further limitation is the use of Nef fused to GFP, since the tag itself might theoretically affect extracellular release. Nevertheless, our complementary experiments demonstrate that h-microglia infected with pseudotyped HIV-1 also secrete Nef-containing EVs, strongly supporting a direct role of Nef in this process. Moreover, previous studies have shown that Nef retains most of its functions when fused to GFP^[69]^.

In summary, across multiple h-microglia HIV-reservoir models, we showed that Nef expression selectively promotes the release of EVs with distinct biophysical and molecular profiles that encapsulate Nef. Our findings provide new insight into the source and characteristics of extracellular Nef in the CNS of HIV-infected individuals and offer a valuable tool for advancing studies of HIV Nef biology.

## DECLARATIONS

### Availability of data and materials

The datasets that validate the original research findings are available from the corresponding author upon reasonable request.

### Copyright

© The Author(s) 2025.
