# Supplementary material for: HIV protein Nef expression in human microglia drives the release of distinct Nef-containing extracellular vesicles
Source: Extracell Vesicles Circ Nucl Acids. 2025 Dec 2;6(4):895–920. doi: 10.20517/evcna.2025.106 (PMC12809690; doi:10.20517/evcna.2025.106)
Supplement: Supplementary file 1 [file evcna-6-4-895-SupplementaryMaterials.pdf]

Supplementary Materials

HIV protein Nef expression in human microglia drives the release of distinct Nef-containing extracellular vesicles

Teja Lavrin[1], Jure Loboda[1], Jana Ferdin[1], Valentina Levak[2,3], Simona Sitar[4], Marija Holcar[1], Nataša Resnik[5], Matjaž Stenovec[6,7], Alenka Trampuš Bakija[8], Peter Veranič [5], Ema Žagar [4], Magda Tušek Žnidarič[2], Pia Pužar Dominkuš[1], Metka Lenassi[1]

[1]Institute of Biochemistry and Molecular Genetics, Faculty of Medicine, University of Ljubljana, Ljubljana 1000, Slovenia.
[2]Department of Biotechnology and Systems Biology, National Institute of Biology, Ljubljana 1000, Slovenia.
[3]Jozef Stefan International Postgraduate School, Ljubljana 1000, Slovenia.
[4]Department of Polymer Chemistry and Technology, National Institute of Chemistry, Ljubljana 1000, Slovenia.
[5]Institute of Cell Biology, Faculty of Medicine, University of Ljubljana, Ljubljana 1000, Slovenia.
[6]Celica Biomedical, Ljubljana 1000, Slovenia.
[7]Laboratory of Neuroendocrinology-Molecular Cell Physiology, Institute of Pathophysiology, Faculty of Medicine, University of Ljubljana, Ljubljana 1000, Slovenia.
[8]Clinical Institute for Special Laboratory Diagnostics, University Children's Hospital, University Medical Centre Ljubljana, Ljubljana 1000, Slovenia.

Correspondence to: Assoc. Prof. Metka Lenassi, Dr. Pia Pužar Dominkuš, Institute of Biochemistry and Molecular Genetics, Faculty of Medicine, University of Ljubljana, Ljubljana 1000, Slovenia. E-mail: metka.lenassi@mf.uni-lj.si; pia.puzar-dominkus@mf.uni-lj.si

S1 Assessment of extracellular vesicle release from immortalized human-microglia (h-microglia) exposed to intracellular Nef.GFP or extracellular ATP or ionomycin

To assess the specificity of the Nef.GFP effect on EV release, we exposed h-microglia to ATP or ionomycin, which are well-established stimulants of EV release [1]. h-microglia cultures at 60–70% confluence were exposed transiently to ATP or ionomycin (both Sigma-Aldrich, USA), or were left undisturbed (control D1) for 24 h. In more detail, before incubation with ATP or ionomycin, cells were washed with DPBS (Sigma-Aldrich, USA), then with sterile-filtered extracellular solution (ECS; 130 mM NaCl, 5 mM KCl, 2 mM CaCl2, 1 mM MgCl2, 10 mM glucose, 10 mM HEPES), and exposed to 1 mM ATP (Sigma-Aldrich, USA) or 2 µM ionomycin in ECS at 37°C for 20 minutes or 10 min, respectively. Afterwards, ECS was replaced with the vesicle-free DMEM with supplements and cells were incubated for another 24 h. EVs were enriched by concentration on 100 kDa membranes, dilution with DPBS and ultracentrifugation at 100,000 × *g*, and analyzed by transmission electron microscopy (TEM) and asymmetric flow field-flow fractionation coupled with multi-angle light-scattering detector (AF4-MALS) (Supplementary Figure 1). TEM analysis indicated the release of larger particles from h-microglia exposed to ATP or ionomycin, compared to the control D1 or Nef.GFP samples (Figure 3B). This was supported by AF4-MALS, which determined the average R_{rms} of 340 nm and 422 nm for ATP and ionomycin, respectively, compared to the average R_{rms} of 172 nm for Nef.GFP (Supplementary Table 1). Additionally, Nef.GFP expression by itself was a stronger inducer of EV release from h-microglia, as AF4-MALS detected lower levels of released particles induced by ATP or ionomycin compared to Nef.GFP sample (2.29 or 5.33 × 10^7 vs. 73.6 × 10^7 particles per million cells; Supplementary Table 1).

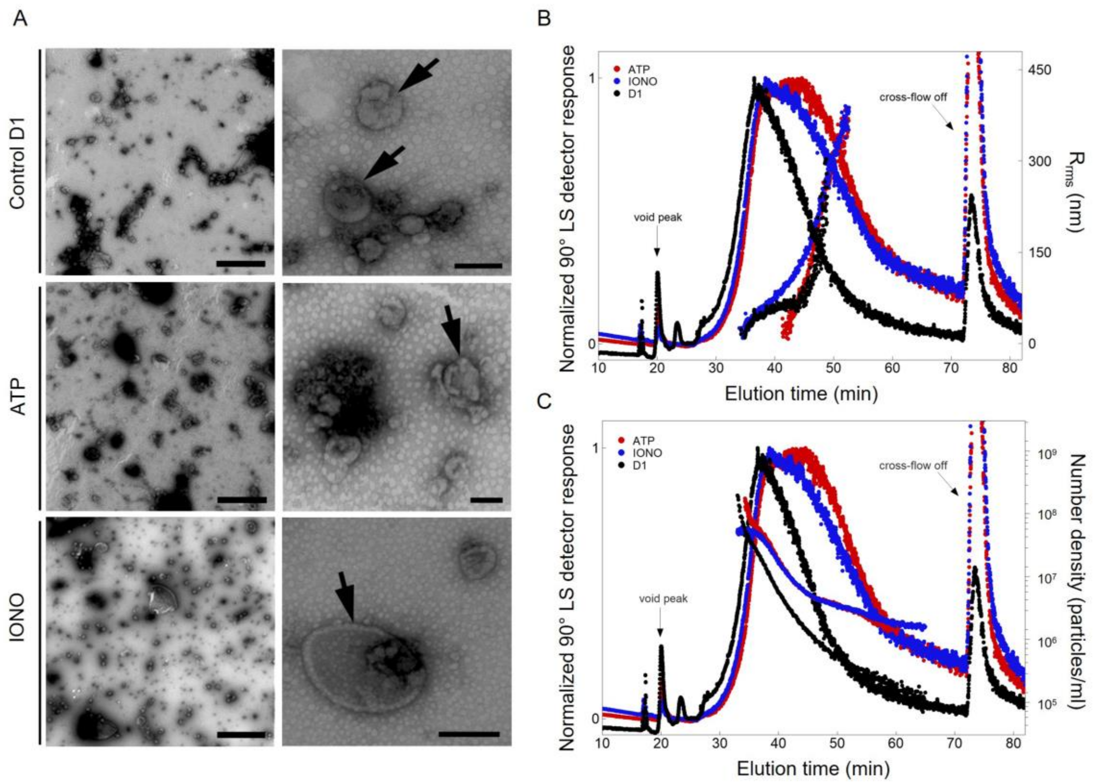

Supplementary Figure 1. ATP and ionomycin stimulate release of larger particles from h-microglia. Crude EVs were enriched by simple ultracentrifugation from media of 24 h old h-microglia cultures exposed to ATP (ATP), ionomycin (IONO) or not (control D1). (A) Representative TEM images of negative-stained pelleted particles of the control D1, ATP and IONO samples. Arrows indicate single vesicular structures. Scale bars: 1 µm (left) and 200 nm (right); (B) Normalized AF4-MALS fractograms recorded by 90° LS detector (solid lines) for pelleted particles of the D1 (black), ATP (red) and IONO (blue) samples, together with root-mean-square radius, R_{rms} (filled circles) as a function of elution time. (C) Normalized AF4-MALS fractograms recorded by 90° LS detector (solid lines) of pelleted particles of the D1 (black), ATP (red) and IONO (blue) samples, together with particle number density per mL (filled circles) as a function of elution time. EVs: extracellular vesicles; TEM: Transmission Electron Microscopy; AF4-MALS: Asymmetric-flow field-flow fractionation coupled to a multi-angle light scattering detector.

Supplementary Table 1. Characterization of particle size (in nm) and number (per million cells) in EV-enriched samples by by AF4-MALS

Sample	Particle No. / million cells	R_{rms} (nm)
Control D1	5.76×10^7	190
Control D2	6.27×10^7	282
Nef.GFP	73.6×10^7	172
ATP	2.29×10^7	340
IONO	5.33×10^7	422

AF4-MALS, Asymmetric-flow field-flow fractionation coupled to multi-angle light scattering detector; R_{rms}, root mean square radius. Control D1: untreated cell culture after 24-h EV collection; Control D2: untreated cell culture after 48-h EV collection; Nef.GFP: cell culture expressing NefSF2-EGFP; ATP: cell culture exposed to ATP for 24 h; IONO: cell culture exposed to ionomycin for 24h.

S2 Characterization of h-microglia cells with stably integrated Nef.GFP transgene under inducible promoter

Using lentiviral vector combined with the TET-ON inducible gene expression system, we established a new h-microglia model with a stably integrated Nef.GFP transgene (LV-Nef.GFP) under a doxycycline (DOX)-inducible promoter (Figure 1). As controls, we prepared h-microglia expressing only the TET-ON 3G transactivator (LV-control) or additionally carrying a DOX-inducible GFP gene (LV-GFP).

To determine the percentage of fluorescent cells after expression activation by 50 ng/mL DOX, we used FACSCantoII Flow Cytometer, equipped with blue and red lasers, and the FACSDiva software (BD Biosciences, USA). Digital data were processed and analyzed with FlowJo software (Tree Star Inc.). After exposure of LV-GFP and LV-Nef.GFP h-microglia cultures to DOX, 98.8% (±2.3%) and 96.6% (±5.0%) of cells expressed GFP and Nef.GFP at 48 hours, respectively (Supplementary Figure 2). h-microglia cultures in the absence of DOX, or LV-control h-microglia exposed to DOX, had negligible levels of background fluorescence (≤ 1.14%). Notably, automatic cell counting after Trypan Blue staining showed that the proportion of dead cells in any experiment was ≤ 5%.

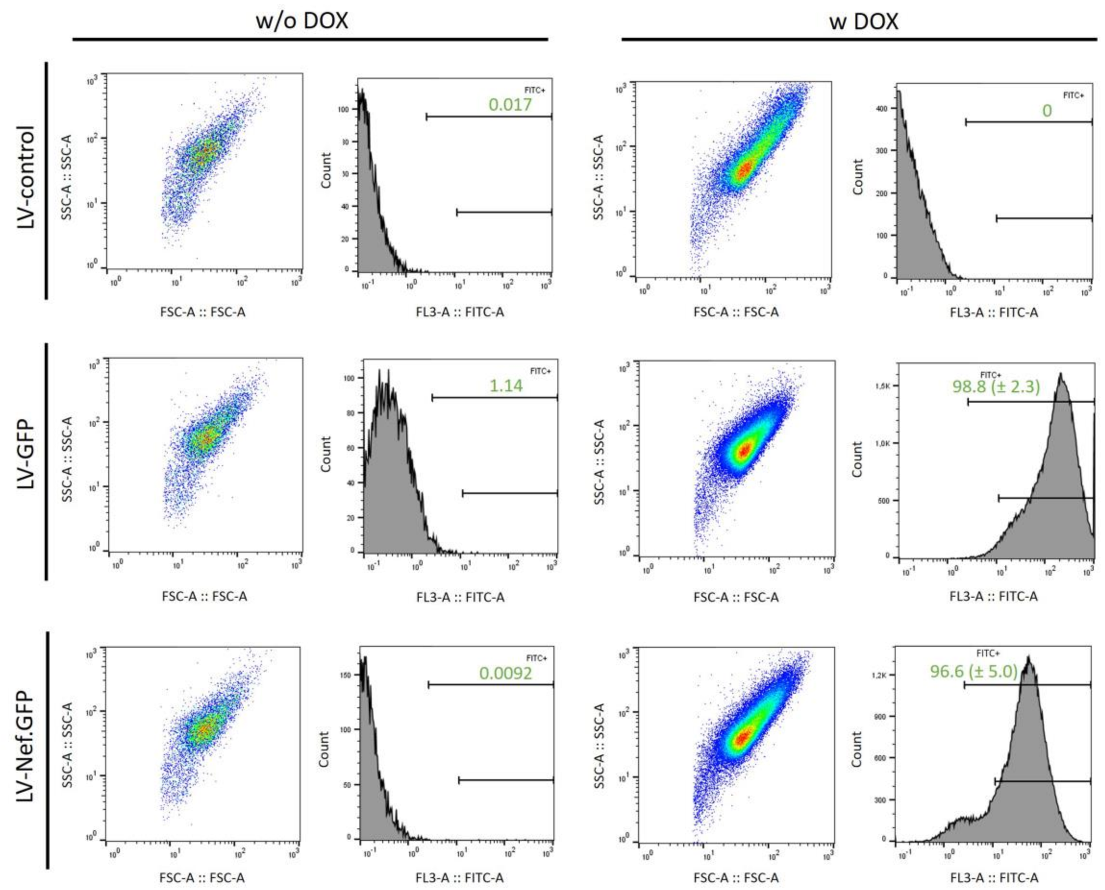

Supplementary Figure 2. h-microglia with stably integrated Nef.GFP transgene express Nef.GFP after exposure to doxycycline. Representative dot plots showing forward scatter (FSC) versus side scatter (SSC) and histograms showing FITC fluorescence intensity for LV-control, LV-GFP and LV-Nef.GFP h-microglia cultures exposed (w) or not (w/o) to 50 ng/mL DOX for 48 h. Percentages (± SD) of $FITC^+$ cells from eight independent experiments, gated on the LV-control population, are indicated on each histogram. Upper line: subset gated on control cells; lower line: subset gated on GFP-positive cells. DOX: doxycycline.

To assess expression of Nef.GFP in h-microglia exposed to DOX over time, we performed live cell imaging with Incucyte S3 Live-Cell Analysis Instrument and Incucyte 2024B GUI software for data collection and processing (both Sartorius AG, Germany). Briefly, h-microglia were seeded at 5×10^3 cells per well in 96-well tissue culture plates (TPP, Switzerland) and grown overnight, after which the media was changed for EV-depleted complete DMEM, supplemented with 50 ng/mL DOX. Images were captured every 15 min in the first two hours after addition of DOX, followed by every two hours until the experimental endpoint at 48 h (see Supplementary

Figure 3). Nef.GFP proteins were visibly expressed in LV-Nef.GFP h-microglia already after six hours, with the percentage of fluorescent cells increasing over time and reaching a plateau (98.2% ± 0.9%) after 36h. All cells in the field of view expressed Nef.GFP. LV-GFP h-microglia similarly responded well to 50 ng/mL DOX, with visible GFP expression starting at two hours. Ninety-six (±1.5%) of cells expressed GFP already after six hours, with the expression persisting until 48 h. Based on their morphology and proliferation rate, the cells appeared healthy as revealed with live cell imaging.

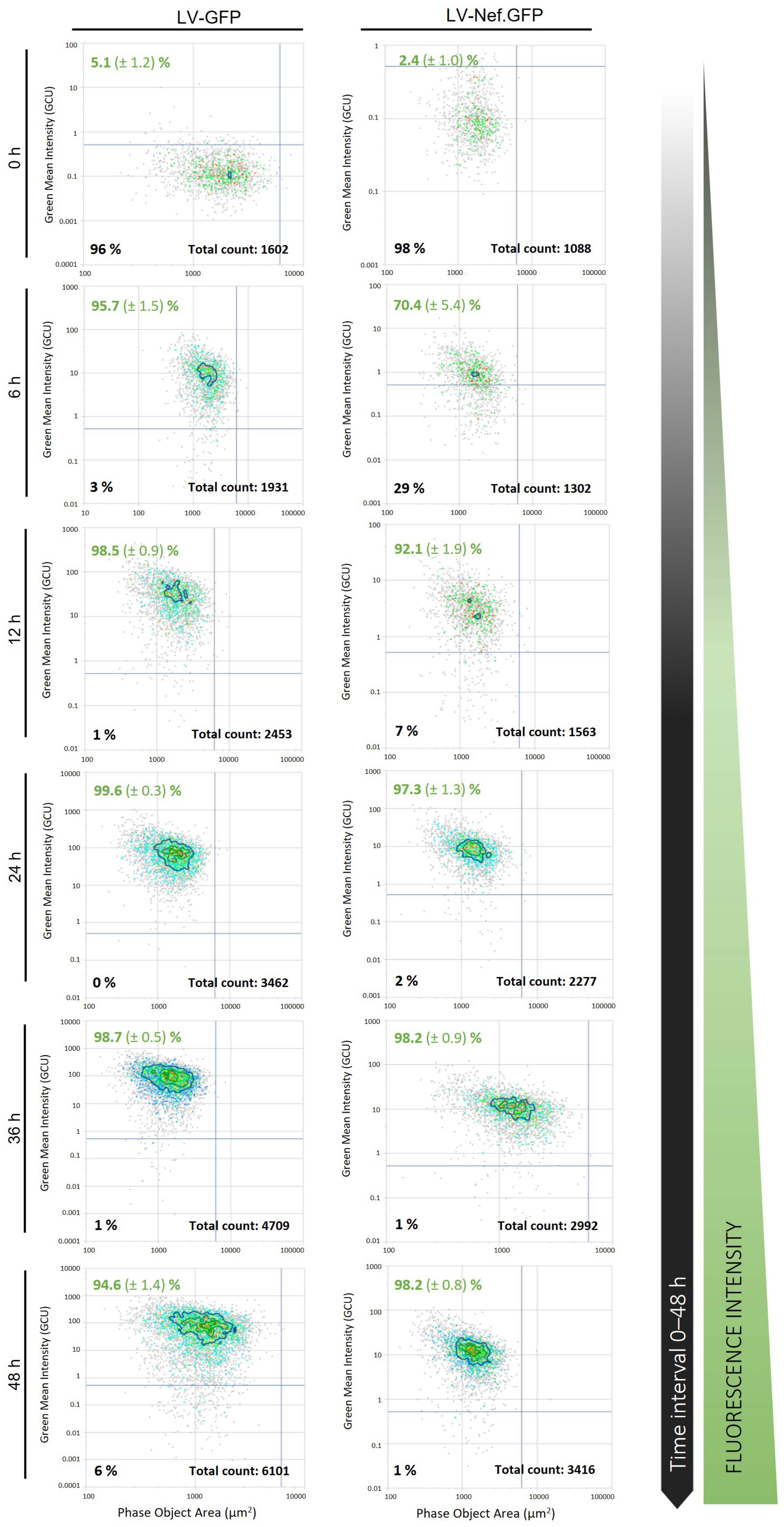

LV-GFP
LV-Nef.GFP
0 h
6 h
12 h
24 h
36 h
48 h
Green Mean Intensity (GCU)
Phase Object Area (µm²)
5.1 (± 1.2) %
96 %
Total count: 1602
2.4 (± 1.0) %
98 %
Total count: 1088
95.7 (± 1.5) %
3 %
Total count: 1931
70.4 (± 5.4) %
29 %
Total count: 1302
98.5 (± 0.9) %
1 %
Total count: 2453
92.1 (± 1.9) %
7 %
Total count: 1563
99.6 (± 0.3) %
0 %
Total count: 3462
97.3 (± 1.3) %
2 %
Total count: 2277
98.7 (± 0.5) %
1 %
Total count: 4709
98.2 (± 0.9) %
1 %
Total count: 2992
94.6 (± 1.4) %
6 %
Total count: 6101
98.2 (± 0.8) %
1 %
Total count: 3416
Time interval 0–48 h
FLUORESCENCE INTENSITY

Supplementary Figure 3. h-microglia with stably integrated Nef.GFP transgene show visible Nef.GFP expression early after doxycycline exposure. Representative dot plots of live cell imaging showing green mean intensity (GCU) versus phase object area (µm2) for LV-GFP and LV-Nef.GFP h-microglia cultures exposed to 50 ng/mL DOX for 48 h. Classification plots were analyzed at time points 0 h, 6 h, 12 h, 24 h, 36 h, and 48 h after onset of treatment. The percentage of GCU-positive cells is indicated within the corresponding quadrants, along with the total cell count included in the analysis (bottom right).

S3 Characterization of Nef.GFP small EVs released from doxycycline-exposed h-microglia with stably integrated Nef.GFP transgene

To further characterize small EVs enriched from LV-GFP and LV-Nef.GFP expressing h-microglia cultures, we separated them over iodixanol density gradient (Optiprep density gradient, ODG) and performed nanoparticle tracking analysis (NTA) of twelve collected fractions. We determined the fraction densities by measuring absorbance at 340 nm (A340) and accounting for the percentage of iodixanol solution for each fraction following the manufacturer's instructions (OptiPrep™ Application Sheet V05) and as previously described [2] (Supplemantary Table 2).

Supplementary Table 2. Density of collected gradient fractions calculated from measured absorbance at 340 nm (A340) and percentage of iodixanol solution for each fraction

Fraction	A_{340}	Fraction density (g/ml)
1	0.065	1.047
2	0.115	1.061
3	0.156	1.072
4	0.198	1.083
5	0.253	1.097
6	0.297	1.109
7	0.402	1.136
8	0.519	1.167
9	0.630	1.196
10	0.964	1.284
11	1.087	1.317
12	1.355	1.388

Fractions 5–9, which were pooled for purified Nef.GFP EV sample, are marked in grey.

We next performed NTA on all collected fractions, but only fractions 6–9 and 6–10 for GFP and Nef.GFP small EV samples, respectively, had ≥10 particles per frame (PPF) for reliable quantification (Supplementary Figure 4, full circles; Supplementary Table 3). Fractions 7–8 and 7–9 for GFP and Nef.GFP small EV samples, respectively, had the highest particle counts expressed as particles per million cells, of comparable mode size.

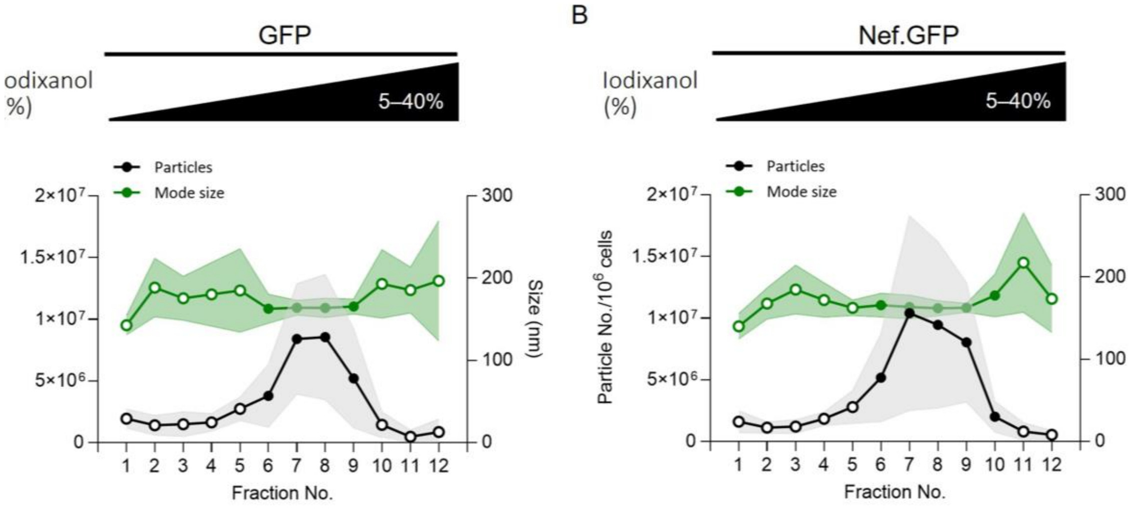

Supplementary Figure 4. Nef.GFP expression from stably integrated Nef.GFP transgene in h-microglia induces the release of EVs. Small EVs enriched from LV-GFP (A) and LV-Nef.GFP (B) culture media were further separated on 5–40% iodixanol density gradient and the twelve collected fractions analyzed by nanoparticle tracking analysis (NTA) for particle number per million cells (black line) and mode size in nm (green line). Particle concentration and mode size values are expressed as mean ± SD from six independent experiments. Filled circles (PPF≥10), empty circles (PPF<10). PPF: particles per frame.

Supplementary Table 3. Characterization of particle size (in nm), number (per million cells) and fluorescence (FITC+) in ODG fractions after separation of EVs by NTA and nano-flow cytometry

EV sample	Fraction No.	NTA (mean ± SD)		Nano-FC (mean ± SD)		
		Particle No./ million cells	Mode size (r in nm)	Particle No./ million cells	$FITC^{+}$ EVs/ million cells	FITC+ (%)
LV-GFP	1	N.A.	N.A.	20.1×10^{6}	5.22×10^{6}	25.9
	2	N.A.	N.A.	19.0×10^{6}	9.03×10^{6}	47.5
	3	N.A.	N.A.	6.66×10^{6}	1.31×10^{6}	19.7
	4	N.A.	N.A.	12.5×10^{6}	0.79×10^{6}	6.4
	5	N.A.	N.A.	9.60×10^{6}	2.13×10^{6}	22.2
	6	$3.80 (\pm 2.56) \times 10^{6}$	162.8 (± 17.9)	20.0×10^{6}	4.47×10^{6}	22.3
	7	$8.41 (\pm 4.49) \times 10^{6}$	164.2 (± 9.1)	43.3×10^{6}	12.0×10^{6}	27.8
	8	$8.55 (\pm 5.10) \times 10^{6}$	163.9 (± 11.9)	46.8×10^{6}	7.82×10^{6}	16.7
	9	$5.21 (\pm 4.02) \times 10^{6}$	165.5 (± 9.3)	15.5×10^{6}	2.16×10^{6}	13.9
	10	N.A.	N.A.	N.A.	N.A.	N.A.
	11	N.A.	N.A.	N.A.	N.A.	N.A.
	12	N.A.	N.A.	N.A.	N.A.	N.A.
LV-Nef.GFP	1	N.A.	N.A.	32.7×10^{6}	0.66×10^{6}	2
	2	N.A.	N.A.	49.8×10^{6}	1.44×10^{6}	2.9
	3	N.A.	N.A.	22.5×10^{6}	1.08×10^{6}	4.8
	4	N.A.	N.A.	39.9×10^{6}	3.03×10^{6}	7.6
	5	$2.79 (\pm 1.33) \times 10^{6}$	162.7 (± 9.7)	71.1×10^{6}	6.12×10^{6}	8.6
	6	$5.18 (\pm 3.57) \times 10^{6}$	165.8 (± 14.6)	77.0×10^{6}	9.01×10^{6}	11.7

7	10.4 (± 7.91) × 10^6	163.7 (± 14.5)	93.9 × 10^6	43.9 × 10^6	46.8
8	9.46 (± 6.57) × 10^6	162.1 (± 9.2)	50.9 × 10^6	12.6 × 10^6	24.8
9	8.04 (± 4.85) × 10^6	162.6 (± 5.5)	103 × 10^6	9.85 × 10^6	9.6
10	2.01 (± 1.25) × 10^6	177.5 (± 26.1)	N.A.	N.A.	N.A.
11	N.A.	N.A.	N.A.	N.A.	N.A.
12	N.A.	N.A.	N.A.	N.A.	N.A.

NTA: Nanoparticle Tracking Analysis; nano-FC: nano-flow cytometer; r: radius; LV-GFP: cell culture stably expressing EGFP; LV-Nef.GFP: cell culture stably expressing NefSF2-EGFP; FITC+: green-fluorescent EVs; N.A.: not applicable (particle per frame <10 and/or fraction density too high). Standard deviations (± SD) for NTA were calculated from three independent experiments. Fractions 5–9, which were pooled for Nef.GFP EVs isolation, are marked in grey.

The first nine of the twelve fractions were also analyzed by nano-flow cytometry to determine particle number per million cells and percentage of fluorescent, GFP or Nef.GFP-positive EVs (Supplementary Table 3). In general, fractions of the separated Nef.GFP small EVs contained 2.9-fold more total and fluorescent particles compared to the fractions of the separated GFP small EVs. For example, fraction seven had 43.9×10^6 vs. 12.0×10^6 fluorescent EVs per million cells for the Nef.GFP sample compared to the GFP sample, respectively. We next pooled ODG fractions 5–9, as these are in the range of typical EV buoyant densities (1.097–1.196 g/mL; Supplementary Table 2) and were positive for typical EV proteins and Nef.GFP (as shown by immunoblotting (Figure 6). We concentrated EVs using Amicon Ultra-15 filters (100 kDa cutoff) and analyzed them again with nano-flow cytometry (Supplementary Figure 5). Up to 35.7% (±3.6%) of EVs were Nef-GFP-positive in the pooled fractions compared to 24.3% (±7.9%) of FITC+ for the GFP sample (73.4×10^6 vs. 56.4×10^6 per million cells, respectively). We observed a slightly higher loss of FITC+ particles after ODG for the LV-Nef.GFP EV sample (approximately 11% retained) compared to the LV-GFP EV sample (approximately 55% retained; Table 1). Nonetheless, this difference in particle loss between the samples did not reach statistical significance ($P > 0.05$, $P = 0.0856$; Kruskal-Wallis test, GraphPad Prism 10.6.0).

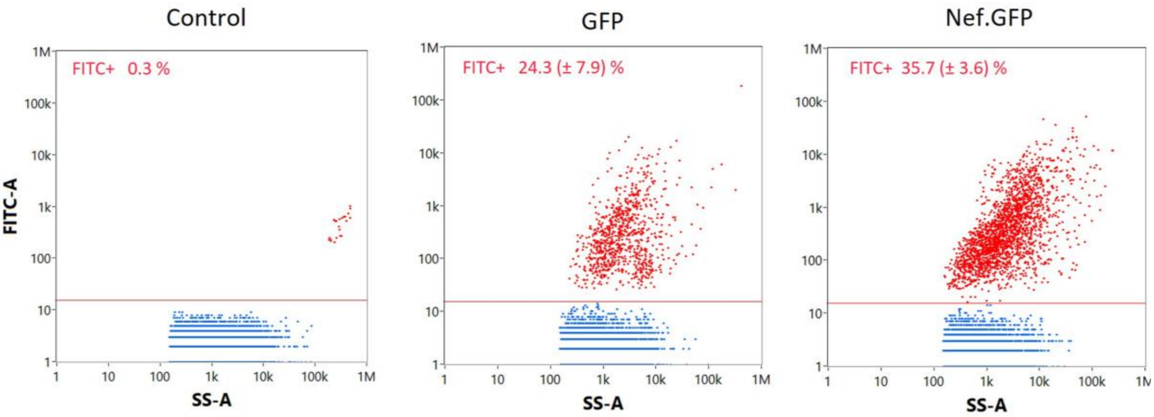

Supplementary Figure 5. Up to half of EVs released from Nef.GFP expressing h-microglia contain detectable Nef.GFP as measured by nano-flow cytometry. EVs were pooled from fractions 5–9 of the iodixanol density gradient after separation of the Nef.GFP small EVs and quantified by nano-flow cytometry. Representative dot plots of pooled EVs from LV-control (Control), LV-GFP (GFP) and LV-Nef.GFP (Nef.GFP) samples are showing forward FITC fluorescence (FITC-A) in relation to the side scatter

(SS-A). GFP+ or Nef.GFP+ EVs (FITC+%) are indicated in red, while non-fluorescent particles are indicated in blue. EV sample enriched from LV-control h-microglia culture was used to gate fluorescent EVs. Respective percentages of fluorescent particles from three independent experiments (± SD) are indicated in the graph.

S4 Nef.GFP is packed inside EVs released from Nef.GFP expressing h-microglia

To support immunogold TEM data showing that Nef.GFP is localized inside EVs (Figure 7), we performed several additional control experiments (Supplementary Figure 6). We included higher magnifications of 0.05% Triton X-100 detergent treated (v-viii) or untreated (i–iv) pooled Nef.GFP EVs, showing pure EV samples with partly degraded vesicles or completely released cargo when treated with detergent. When we performed immunogold labelling against Nef on detergent-untreated Nef.GFP EVs (i-iv), rare individual gold nanoparticles were visible (white arrows), but did not appear bound to intact EVs, indicating absence of detectable Nef on the surface of EVs. Importantly, the specificity of Nef labeling is supported by the absence of gold nanoparticles in detergent-treated Nef.GFP EVs subjected to immunogold labeling without inclusion of primary antibodies against Nef.GFP (v).

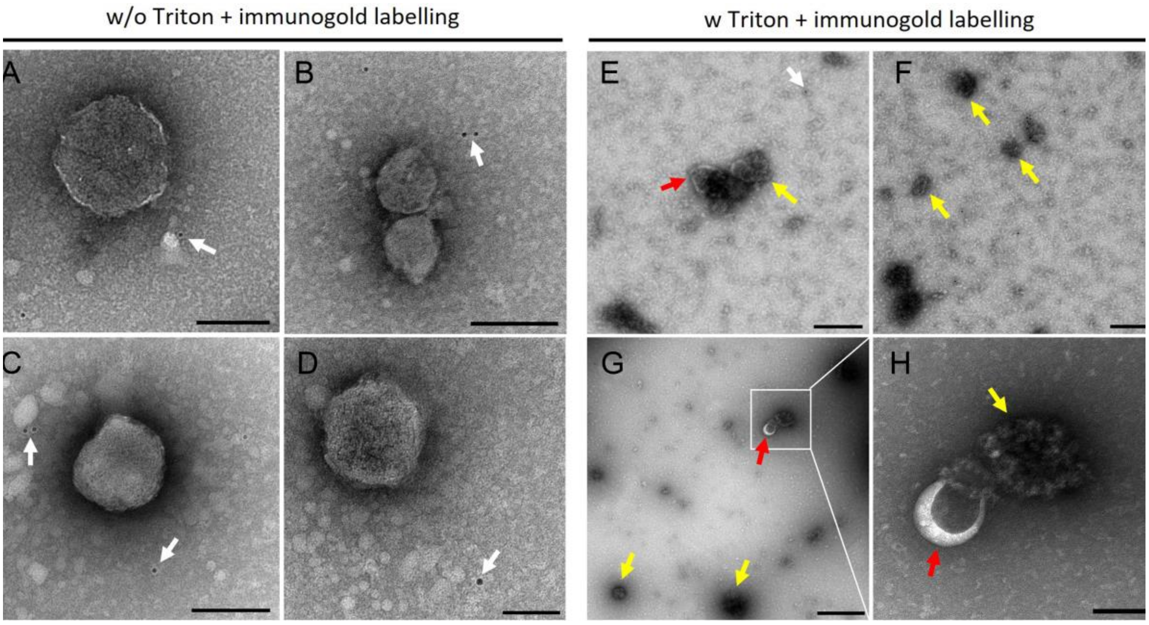

Supplementary Figure 6. Control experiments for TEM analysis of Nef.GFP EVs after immunogold labelling against Nef. EVs from fractions 5–9 of gradient separated Nef.GFP EV-enriched sample were concentrated and analyzed by immunogold TEM. (i-iv) Anti-Nef immunogold labelling of Nef.GFP EVs not treated with detergent showing intact vesicles; rare individual gold nanoparticles were visible but were not bound to EVs (white arrows). (v) The overview of the detergent-treated Nef.GFP EVs immunolabelled with IgG (control of procedure) alone showing no or only a few gold nanoparticles (white arrow). (vi) Not all released cargo (pointed with yellow arrows) was labelled with gold nanoparticles after anti-Nef immunolabelling. (vii) The overview of Nef.GFP EVs treated with Triton X-100, which shows partly degraded vesicle (pointed with red arrow)

enlarged in (viii) or completely collapsed vesicles and released cargo (yellow arrows). Gold nanoparticles are pointed with white arrow. Scale bars: 100 µm (iv,viii), 200 µm (i-iii, v-vi), 500 µm (vii); TEM: Transmission Electron Microscopy

REFERENCES

1. Lombardi M, Gabrielli M, Adinolfi E, Verderio C; Role of ATP in Extracellular Vesicle Biogenesis and Dynamics. *Front Pharmacol* 2021;12. DOI: https://doi.org/10.3389/fphar.2021.654023

2. Van Deun J, Mestdagh P, Sormunen R, Cocquyt V, Vermaelen K, et al. The impact of disparate isolation methods for extracellular vesicles on downstream RNA profiling. *J Extracell Vesicles* 2014;3(1) PMID: 25317274 DOI: https://doi.org/10.3402/jev.v3.24858
